# Systemic Propagation of STING Signalling via Generation of Large Extracellular Vesicles

**DOI:** 10.1002/jev2.70289

**Published:** 2026-05-09

**Authors:** Jiae Lee, Annabel Vernon, Hyung Joon Park, Young V. Kwon

**Affiliations:** ^1^ Department of Biochemistry University of Washington Seattle Washington USA; ^2^ Department of Biological Sciences California State University Long Beach Long Beach California USA

**Keywords:** cancer, Drosophila, extracellular vesicles, immune, inflammation, JNK, STING

## Abstract

Large extracellular vesicles (EVs) derived from tumour cells play important roles in tumour formation and progression. However, it remains unclear why malignant cells produce these EVs and how they act in vivo. We employ a well‐characterized *Drosophila* tumour model and demonstrate that large EV biogenesis from malignant cells is an evolutionarily conserved process. Our study uncovers an essential role for the cyclic GMP‐AMP synthase (cGAS)‐stimulator of interferon genes (STING) pathway, which mediates the innate immune response to cytosolic DNA, in driving large EV biogenesis and inducing a systemic immune response to tumours. STING engages a signalling axis comprising JNK and FAK—independent of TANK‐binding kinase 1 (TBK1) and inhibitor of nuclear factor κB kinase (IKKβ)—to drive large EV biogenesis in both *Drosophila* and human malignant cells. Transplantation of large EVs from *Drosophila* tumours to wild type larvae is sufficient to recapitulate the systemic immune response to tumours by activating STING signalling in macrophage‐like cells. Thus, our study establishes a novel animal model for studying large EVs derived from malignant cells and provides insights into how STING signalling propagates from tumour cells to the immune system via large EV biogenesis, inducing a systemic immune response to tumours.

## 1 Introduction

Cells produce heterogeneous extracellular vesicles (EVs) to communicate with cells nearby and at a distance by transferring bioactive cargo such as proteins, nucleic acids, lipids, and metabolites (Kalluri and LeBleu 2020; Meehan et al. 2016; Salomon et al. 2022; Sheehan and D'Souza‐Schorey 2019; van Niel et al. 2018; van Niel et al. 2022). EVs play versatile roles in numerous biological processes, including immunity, development, blood coagulation, tissue repair, and stem cell maintenance (Koniusz et al. 2016; S et al. 2013; Yanez‐Mo et al. 2015). Moreover, EVs have been implicated in multiple diseases, including cardiovascular diseases, diabetes, neurodegenerative diseases, and cancers (Choi et al. 2017; Grange and Bussolati 2022; Hu et al. 2023; Kalluri and LeBleu 2020; Minciacchi et al. 2015; Patras et al. 2023; Sheehan and D'Souza‐Schorey 2019). EVs can be further defined based on their size and production mechanism (Meehan et al. 2016; Yanez‐Mo et al. 2015). Exosomes are around 100 nm in diameter and are primarily produced in the endosomal system (Kalluri and LeBleu 2020). Microvesicles are produced by membrane budding and have diameters typically ranging from 50 nm to 1 µm (van Niel et al. 2018). EVs even larger than 1 µm have been described (Headley et al. 2016; Meehan et al. 2016; van Niel et al. 2018). Tumour cells produce multiple types of EVs, including those larger than conventional microvesicles, which are known as large oncosomes and cytoplasts (Headley et al. 2016; Meehan et al. 2016; van Niel et al. 2018).

Tumour cell‐derived EVs have been shown to play key roles in tumour formation, progression, and metastasis by transporting oncogenic macromolecules, modifying the tumour microenvironment, and communicating with immune cells (Choi et al. 2017; Hu et al. 2023; Minciacchi et al. 2015; Minciacchi et al. 2017; Patras et al. 2023; Sheehan and D'Souza‐Schorey 2019). Since EVs are typically much smaller than cells, observing them in action in a physiological context is challenging. Moreover, genetic studies of EVs often require the manipulation of multiple cell types as EVs can be a means to communicate with recipient cells nearby or even in different tissues. A simple genetic model allowing observation of EVs in action and robust genetic manipulation of their donor and recipient cell types would be useful for studying EV biology. Considering the advanced genetic tools available and the transparency of the animal, *Drosophila* larvae provide a favourable platform for studying EVs in their physiological context. *Drosophila* have been used for studying exosome‐like EVs in synaptic growth during development, Wingless (Wg) and Hedgehog (Hh) signalling during wing development, and the reprogramming of female behaviour after mating (Gross et al. 2012; Parchure et al. 2018). However, large EVs, such as tumour‐derived large EVs, have not been clearly described in *Drosophila*. We previously observed that oncogenic *Ras* (*Ras^V12^*)‐transformed intestinal epithelial cells produce EVs larger than 1 µm (Lee et al. 2020). In this study, we demonstrate that similar large EVs are robustly produced from the well‐characterized malignant epithelial tumours, generated by expressing *Ras^V12^* and deleting the polarity gene *scribble* (*scrib*) (*Ras^V12^*, *scrib^−/−^* clones) (Pagliarini and Xu 2003).

Tumours interact with immune cells, inducing either anti‐tumour or pro‐tumour immune responses depending on the context (Furumaya et al. 2020; Ostrand‐Rosenberg 2008; Zhao et al. 2021). Similarly, *Drosophila* tumours induce immune responses, which suppress or enhance tumour growth and progression (Cordero et al. 2010; Parisi et al. 2014; Pastor‐Pareja et al. 2008). *Ras^V12^*, *scrib^−/−^* clones in eye‐antennal discs grow into large masses, which can metastasize to distant tissues (Pagliarini and Xu 2003). *Ras^V12^*, *scrib^−/−^* tumours recruit haemocytes—macrophage‐like cells—and increase the number of circulating haemocytes possibly by allowing them to divide more (Pastor‐Pareja et al. 2008). Moreover, haemocytes produce the *Drosophila* tumour necrosis factor (TNF) Eiger in response to *Ras^V12^*, *scrib^−/−^* tumours, which unexpectedly promotes tumour growth (Igaki et al. 2009; Mangoni et al. 2016; Parisi et al. 2014). The *Drosophila* adipose tissue, the fat body, is also an immune tissue and secretes antimicrobial peptides and inflammatory cytokines into the haemolymph in response to infection, tissue damage, and oncogenic transformation of epithelia (Ferrandon et al. 2007; Hauling et al. 2014; Parisi et al. 2014). Interestingly, *Ras^V12^*, *scrib^−/−^* tumours induce the expression of the antimicrobial peptide Drosomycin (Drs) in the fat body in a manner dependent on haemocytes (Parisi et al. 2014). Immune signalling in the fat body also impacts tumour growth in the imaginal discs (Parisi et al. 2014). Therefore, the interaction between tumours and the immune system in *Drosophila* involves multiple tissues and induces localized as well as systemic immune responses. These findings suggest that the communication between tumours and the host immune system is a complex process. The recruitment of haemocytes to tumours and the engulfment of tumour cell fragments by haemocytes might be important for the communication between tumours and the host immune system (Cordero et al. 2010; Hauling et al. 2014; Parisi et al. 2014). However, it is not well studied how haemocytes are recruited to tumours and whether tumour cell fragments are sufficient for inducing an immune response to tumours. In mammals, tumour‐derived EVs play versatile roles in the communication between tumours and immune cells. Whether these roles are conserved in *Drosophila* remains to be determined.

In humans, the cyclic GMP‐AMP synthase (cGAS)‐stimulator of interferon genes (STING) signalling pathway mediates an innate immune response to cytosolic DNA originating from various sources (Cai et al. 2014; Decout et al. 2021; Hopfner and Hornung 2020). cGAS is activated by dsDNA produced from bacterial or viral infection, chromosomal instability, or mitochondrial damage (Chen et al. 2016; Ding et al. 2025; Lai et al. 2025). Upon binding cytosolic DNA, cGAS undergoes liquid‐phase condensation and produces the second messenger cyclic GMP‐AMP (cGAMP) (Du and Chen 2018; Hopfner and Hornung 2020). In turn, cGAMP binds to STING in the endoplasmic reticulum (ER) membrane, which causes STING to oligomerize and translocate to the Golgi. There, STING recruits and activates TANK‐binding kinase 1 (TBK1), which then phosphorylates interferon regulatory factor 3 (IRF3), leading to an induction of downstream genes, including type I interferons. Additionally, STING activates IκB kinase (IKK) to induce the expression of NF‐κB‐mediated inflammatory genes (Hopfner and Hornung 2020). Thus, the cGAS‐STING signalling pathway could play a key role in the immune response to tumours by cell‐autonomously inducing type I interferons and communicating with immune cells in the microenvironment (Hopfner and Hornung 2020; Kwon and Bakhoum 2020). EVs, such as exosomes and apoptotic blebs, are proposed to carry tumour DNA, which can be imported into immune cells to activate STING (Hopfner and Hornung 2020; Kwon and Bakhoum 2020). Chromosomal instability (CIN) is a hallmark of cancers (Kwon and Bakhoum 2020). CIN is proposed to be the primary source of cytosolic DNA in cancer cells (Kwon and Bakhoum 2020). Thus, cytosolic DNA in tumour cells is likely to be the primary source of tumour DNA in EVs. Although the cGAS‐STING pathway responds to cytosolic DNA, it is not currently known whether cGAS‐STING signalling drives the biogenesis of tumour‐derived EVs, particularly those that carry tumour DNA.

The cGAS‐STING pathway is also present in insects, including *Drosophila* (Cai et al. 2022). *Drosophila* STING and the cGAS‐like receptors (cGLRs) function in the immune response to infection by viruses, such as *Drosophila* C virus (DCV), Cricket Paralysis virus (CrPV), and Zika virus (Goto et al. 2020; Martin et al. 2018). Although *Drosophila* does not encode type I interferons, activation of *Drosophila* STING induces the expression of antimicrobial peptides, which constitute a humoral immune response to infection, as well as a set of STING‐dependent antiviral response genes (Goto et al. 2020; Martin et al. 2018). Interestingly, recent studies have shown that CIN is associated with tumour formation and cell invasion in *Drosophila* (Benhra et al. 2018; Wang et al. 2021). However, cGAS‐STING signalling has not been studied in the context of tumours in *Drosophila*. Whether cGAS‐STING signalling might be also elevated in *Drosophila* tumours remains unknown.

In this study, we employ a well‐characterized malignant epithelial tumour model and demonstrate that the biogenesis of large EVs from malignant cells is a conserved process in *Drosophila*, and that these EVs are readily traceable in their native context. Notably, our study discovers that STING engages a signalling axis independent of TBK1 and IKK to induce large EV biogenesis from both *Drosophila* and human malignant cells. Transplantation of these large EV fractions into wild‐type larvae is sufficient to induce a systemic immune response in a manner dependent on STING in haemocytes. Altogether, we establish *Drosophila* as an attractive animal model for studying tumour‐derived large EVs in their physiological environment and describe how a cytosolic DNA response propagates from tumour to host immune system by driving large EV biogenesis to induce a systemic immune response to tumours.

## 2 Results

### 2.1 The Generation of Large EVs From Malignant Cells is an Evolutionarily Conserved Process

**FIGURE 1:**
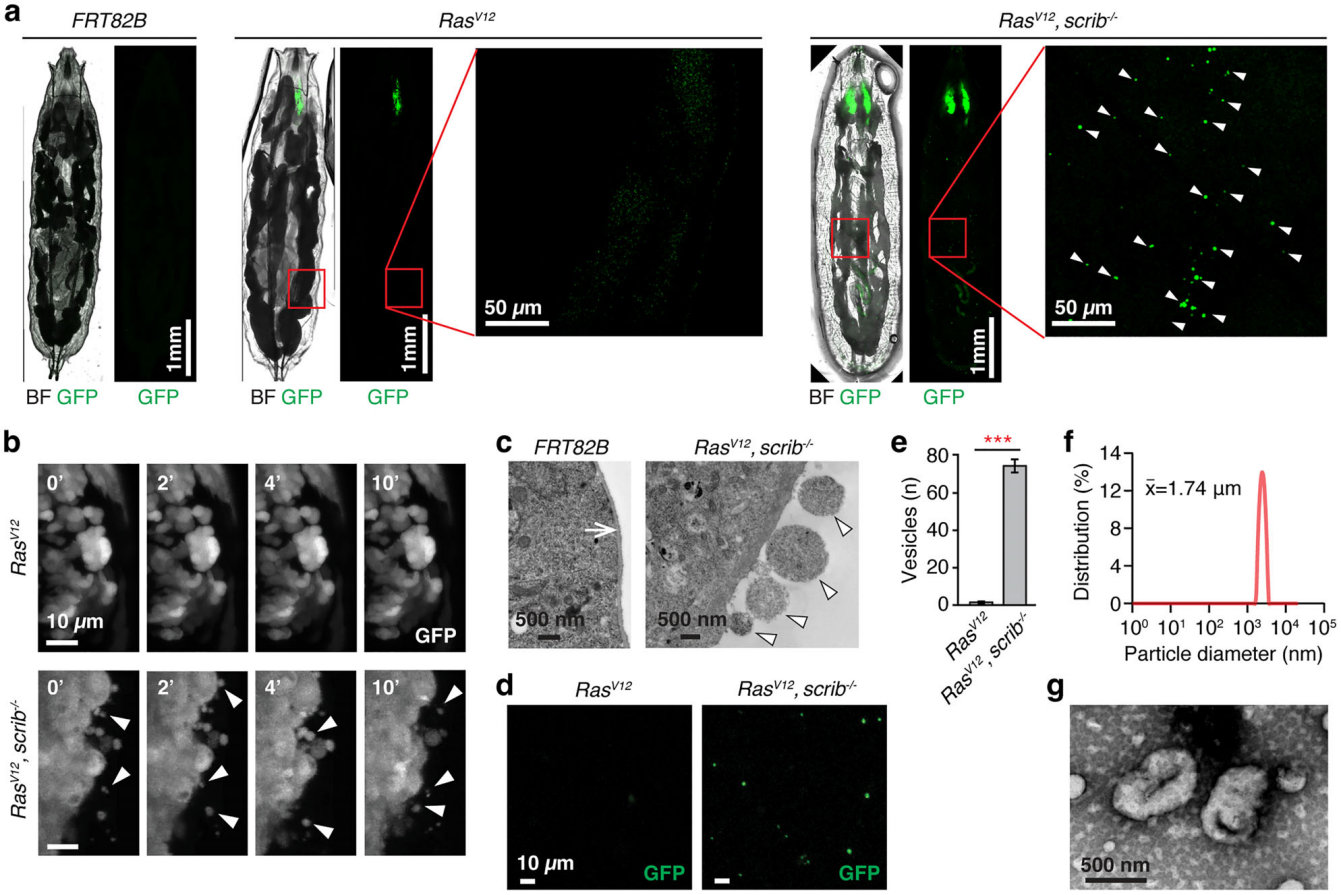
***Drosophila* eye‐antennal disc *Ras^V12^, scrib^−^^/^^−^*** tumour**s generate large EVs**. (a) Confocal tile‐scan images of live whole mounted *FRT82B* control (6 d AEL), *Ras^V12^* (6 d AEL), and *Ras^V12^, scrib^−/−^* (8 d AEL) larvae. Arrowheads indicate GFP‐positive particles detected inside larvae. GFP is expressed in clones in eye discs. BF, brightfield. (b) Still shots from *ex vivo* live imaging of eye disc clones. Arrowheads indicate blebs and EVs. Scale bar, 10 µm. Full movies can be found as Videos S3 and S4. (c) Electron microscope images of *Ras^V12^* (6 d AEL) and *Ras^V12^, scrib^−/−^* (8 d AEL) eye discs. Arrow points to the extracellular matrix present in a control disc, and arrowheads indicate EV‐like entities on the surface of a *Ras^V12^, scrib^−/−^* disc. (d) Confocal microscopy of the large EV‐enriched fractions isolated from *Ras^V12^* (6 d AEL) and *Ras^V12^, scrib^−/−^* (8 d AEL) eye discs. (e) Quantification of large EVs generated from *Ras^V12^* (6 d AEL) and *Ras^V12^, scrib^−/−^* (8 d AEL) eye discs *ex vivo*. The number of large EVs was measured at 2 h post incubation. *N* = 3 independent experiments for each genotype. Mean ± SEM are shown. *p < 0.01, Student's t‐test. (f) Diameter of EVs produced from *Ras^V12^, scrib^−/−^* discs. The mean diameter of these EVs is 1.74 µm. (g) Electron microscopy of the isolated EVs.

In *Drosophila*, *Ras^V12^*, *scrib^−/−^* mitotic clones in larval eye discs undergo malignant transformation and acquire the ability to metastasize, whereas clones that express *Ras* ^*V12*^ alone fail to do so (Pagliarini and Xu 2003; Wu et al. 2010) (Figure S1). When these clonal cells were marked by GFP, freely floating GFP^+^ particles were detected inside the larvae carrying *Ras^V12^*, *scrib* ^−/−^ clones, but not inside larvae carrying either wild‐type or *Ras^V12^* clones (Figure 1a; Videos S1 and S2).

To address whether these GFP^+^ particles were indeed produced from *Ras^V12^*, *scrib* ^−/−^ discs, we imaged the discs *ex vivo*. *Ras^V12^*, *scrib* ^−/−^ clones frequently shed off GFP^+^ particles while *Ras^V12^* clones rarely produced them (Figure 1b and Videos S3 and S4). Next, we attempted to visualize these particles using electron microscopy (EM). A layer of homogenous EM signal, which presumably represents the extracellular matrix (ECM), was detected at the surface of wild‐type discs (Figure 1c, arrow). In contrast, the ECM‐like layer was significantly compromised in *Ras^V12^*, *scrib* ^−/−^ discs (Figure 1c). Moreover, we detected round vesicle‐like entities at the surface of *Ras^V12^*, *scrib* ^−/−^ discs (Figure 1c, arrowheads). These entities were generally larger than 500 nm but smaller than 2 µm. The contents appeared to be homogeneous, and nuclei were not found (Figure 1c), indicating that they were not disseminating disc epithelial cells.

Next, we attempted to produce GFP^+^ particles *ex vivo* from *Ras^V12^* or *Ras^V12^*, *scrib* ^−/−^ discs and enrich them by differential centrifugation (Figure 1d; see Materials and Methods). We detected significantly more GFP^+^ particles in the fractions enriched from *Ras^V12^*, *scrib* ^−/−^ discs compared to those from *Ras^V12^* discs (Figure 1e). These particles were approximately 1.7 µm in diameter (Figure 1f). EM of the enriched fractions revealed particles adopting a deflated ball‐like shape (Figure 1g), reminiscent of large EVs previously imaged (Akers et al. 2016; Di Vizio et al. 2012).

These characteristics closely resemble those of large EVs produced from cancer cells (Choi et al. 2017; Headley et al. 2016; Jansen et al. 2019; Meehan et al. 2016; Minciacchi et al. 2015). Thus, the generation of large EVs from malignant cells is an evolutionarily conserved process, establishing *Drosophila* as a novel animal model for studying tumour‐derived large EVs.

### 2.2 Large EVs Induce a Systemic Immune Response in a Haemocyte‐Dependent Manner

**FIGURE 2:**
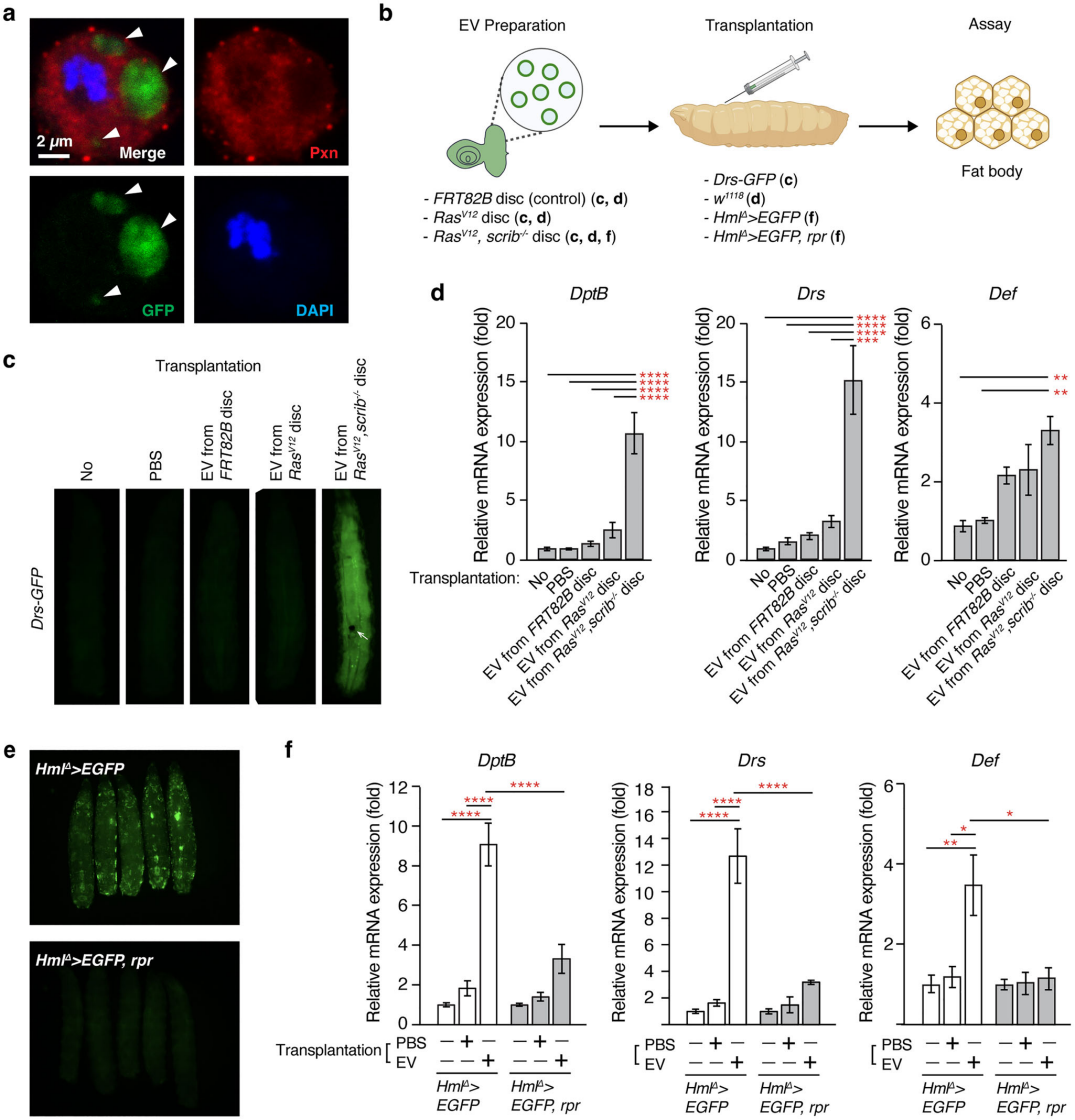
**Large EV fractions obtained from *Ras^V12^, scrib^−^^/^^−^* discs are sufficient to induce a systemic immune response in a manner dependent on haemocytes**. (a) Representative image of a GFP^+^ particle‐internalized by a haemocyte from a larva with *Ras^V12^, scrib^−/−^* discs. Arrowheads point to *GFP^+^* particles. Plasmatocytes are labelled with anti‐Peroxidasin (Pxn) antibody staining. (b) Schematic of large EV transplantation experiment. Large EV fractions are prepared from *FRT82B* control (6 d AEL), *Ras^V12^* (6 d AEL), and *Ras^V12^, scrib^−/−^* (8 d AEL) discs and then transplanted into early 3^rd^ instar larvae with desired genotypes. To eliminate haemocytes, Reaper (Rpr) is expressed with *Hml^∆^‐GAL4*. To assess immune responses, the expression of antimicrobial peptides in fat body was assayed by *Drs‐GFP* and qRT‐PCR. (c) Fluorescent microscopy of live whole‐mount larvae. Early 3^rd^ instar *Drs‐GFP* larvae were transplanted with PBS or large EV fractions prepared from *FRT82B*, *Ras^V12^*, or *Ras^V12^, scrib^−/−^* eye discs. No transplantation was included as a control. Arrow indicates the melanized scab formed from transplantation. Larvae were imaged at 18 h post‐transplantation. (d) Relative mRNA expression of antimicrobial peptides in the fat body after transplantation with PBS or large EV fractions prepared from the indicated eye discs. Mean ± SEM are shown. **p < 0.01, ***p < 0.001, ****p < 0.0001, one‐way ANOVA. (e) Fluorescent microscope images of live whole‐mount larvae. (f) Relative mRNA expression of antimicrobial peptides in the fat body after transplantation with PBS or large EV fractions prepared from the indicated eye discs. Mean ± SEMs are shown. *p < 0.05, **p < 0.01, ****p < 0.0001, one‐way ANOVA.

EVs transduce signals by transporting bioactive cargo to recipient cells (Choi et al. 2017; S et al. 2013). We observed that haemocytes engulfed GFP^+^ particles, reminiscent of large EVs derived from *Ras^V12^, scrib^−/−^* discs (Figure 2a). Thus, we attempted to address the role of these EVs in communication between tumours and the host immune system. We confirmed that *Ras^V12^, scrib^−/−^* tumours increased the expression of the antimicrobial peptide *Drs* in the fat body (Parisi et al. 2014) (Figure S2). Moreover, we found that *Ras^V12^, scrib^−/−^* discs also induced the expression of several additional antimicrobial peptide genes, such as *Diptericin B* (*DptB*), *Defensin* (*Def*), *Attacin‐A* (*AttA*), *Metchnikowin* (*Mtk*), and *Drosocin* (*Dro*), in the fat body (Figure S2). These observations indicate that *Ras^V12^, scrib^−/−^* tumours induce a robust systemic immune response.

Given the observation that GFP^+^ particles resembling large EVs were engulfed by haemocytes, we hypothesized that large EVs might be a signalling modality for the communication between tumours and host immune cells. To address whether large EVs derived from *Ras^V12^, scrib^−/−^* tumours could increase the expression of antimicrobial peptides in the fat body, we transplanted large EV fractions prepared from *Ras^V12^, scrib^−/−^* tumours to *Drs‐GFP* reporter larvae, which express GFP under the control of the *Drs* promotor (Ferrandon et al. 1998) (Figure 2b,c). As controls, we prepared un‐transplanted, phosphate buffered saline (PBS)‐transplanted (mock), wild‐type disc‐derived EV fraction‐transplanted, and *Ras^V12^* disc‐derived EV fraction‐transplanted larvae. GFP signals in the mock‐transplanted animals were indistinguishable from those in the un‐transplanted animals (Figure 2c). Moreover, transplantation of the EV fractions from wild‐type or *Ras^V12^* discs did not increase *Drs‐GFP* signal (Figure 2c). In contrast, transplantation of the EV fractions from *Ras^V12^, scrib^−/−^* discs resulted in an evident increase in GFP signal (Figure 2c). To further characterize the large EV‐induced immune response, we chose three antimicrobial peptides, which represent the main categories of immune signalling pathways (Hanson et al. 2019). Transplantation of PBS alone (mock) or EV fractions prepared from either wild‐type (*FRT82B*) or *Ras^V12^* discs did not significantly alter the mRNA expression of *DptB*, *Drs*, or *Def* in the fat body (Figure 2b,d). In contrast, transplantation of EV fractions prepared from *Ras^V12^, scrib^−/−^* discs significantly increased the mRNA expression of all three antimicrobial peptides (Figure 2d). Injection of either large EV wash fractions or small EVs derived from *Ras^V12^, scrib^−/−^* discs had no effect on AMP expression (Figure S3). Together, these results demonstrate that large EV fractions prepared from *Ras^V12^, scrib^−/−^* discs are sufficient to increase the expression of antimicrobial peptides, which constitutes the systemic immune response to *Ras^V12^, scrib^−/−^* tumours.

Next, we addressed the requirement of haemocytes for the immune response by genetically eliminating them (Figure 2b). Expression of the cell death inducer Reaper (Rpr) with the haemocyte‐specific GAL4 driver (*Hml^Δ^‐GAL4*) depleted haemocytes, which were marked by eGFP (Figure 2e). Transplantation of EV fractions prepared from *Ras^V12^, scrib^−/−^* discs significantly increased the expression of *DptB*, *Drs*, and *Def* in the fat body of wild‐type larvae (*Hml^Δ^* >*EGFP*) when compared to un‐transplanted or PBS‐transplanted larvae (Figure 2f). Genetic ablation of haemocytes (*Hml^Δ^* >*EGFP, rpr*) reversed this increase in the mRNA expression of *DptB*, *Drs*, and *Def* in the fat body (Figure 2f). Thus, haemocytes are required for the systemic immune response induced by large EVs.

### 2.3 Features of Chromosomal Instability Prevail, and STING Signalling is Elevated in *Ras* ^*V12*^, *scrib* ^*−/−*^ Disc Tumours

**FIGURE 3:**
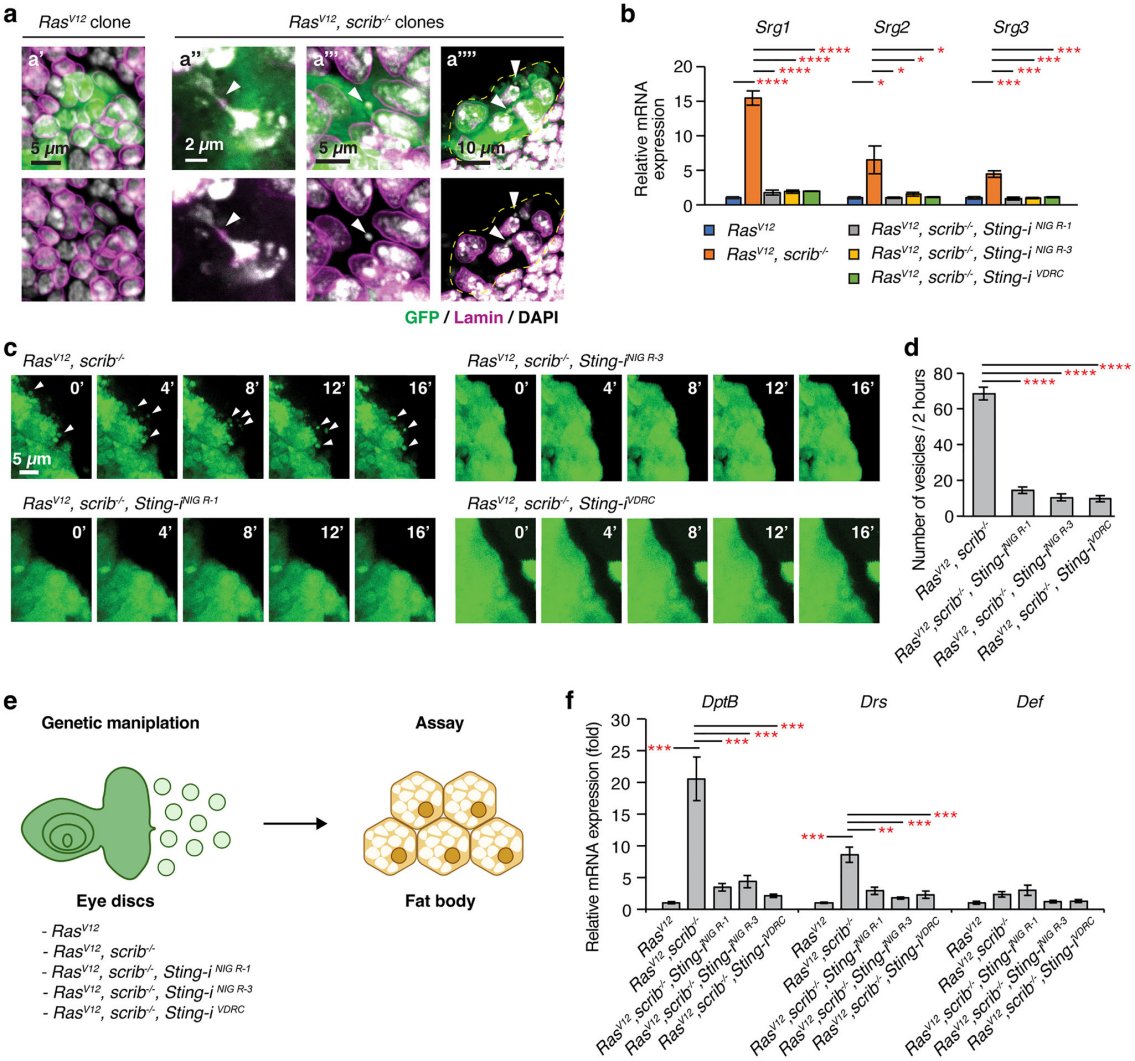
**STING in *Ras^V12^, scrib^−^^/^^−^* clones plays a key role in controlling the production of large EVs**. (a) Images of eye discs with *Ras^V12^* (a’) or *Ras^V12^, scrib^−/−^* clones (a“‐a”″). Arrowheads indicate the features associated with CIN: a“: chromatin bridge, a”’: micronucleus, and a“”: cytosolic leakage of chromatin. Clonal cells are marked with GFP (green), the nuclear envelope is stained with anti‐Lamin antibody (magenta), and DNA is stained with DAPI (grey). (b) Relative mRNA expression of STING target genes in eye‐antennal discs. *Ras^V12^* discs were prepared at 6 d AEL. Other discs were prepared at 8 d AEL. Mean ± SEMs are shown. *p < 0.05, **p < 0.01, ***p < 0.001, ****p < 0.0001, one‐way ANOVA. (c) Still shots from *ex vivo* live imaging of eye discs. Arrowheads indicate blebs and EVs. See Videos S5–S8 for full movies. (d) Quantification of EVs generated from eye discs from 8 d AEL larvae. EVs were quantified at 2 h post incubation. *N* = 3 for each genotype. Mean ± SEMs are shown. *p < 0.05, **p < 0.01, ***p < 0.001, ****p < 0.0001, one‐way ANOVA. (e) Schematic of experiments used to assess the expression of antimicrobial peptides in the fat body upon manipulation of *Sting* in eye disc *Ras^V12^, scrib^−/−^* clones. (f) Relative mRNA expression of antimicrobial peptides in the fat body. Fat body from larvae with *Ras^V12^* eye disc clones was prepared from 6 d AEL larvae. Fat body from other genotypes was prepared from 8 d AEL larvae. Mean ± SEMs are shown. **p < 0.01, ***p < 0.001 one‐way ANOVA.

We leveraged our observations in *Drosophila* to gain insights into the mechanism driving large EV biogenesis from malignant cells. Given that the generation of large EVs is commonly observed in various cancer cells and even in *Drosophila* malignant cells, their occurrence might be associated with a fundamental feature of malignancy. Chromosomal instability (CIN) is a hallmark of cancers (Bakhoum and Cantley 2018; Samson and Ablasser 2022). Cytosolic DNA associated with CIN activates the cGAS‐STING pathway, which plays a crucial role in tumour immunity and progression (Ablasser and Chen 2019; Bakhoum et al. 2018; Mackenzie et al. 2017; Samson and Ablasser 2022). Nevertheless, the function of the cGAS‐STING pathway in large EV biogenesis is unknown. Interestingly, multiple features associated with CIN were observed in *Ras^V12^, scrib^−/−^* clones (Figure 3a″‐a″″, GFP^+^ cells), but not in *Ras^V12^* clones (Figure 3a’, GFP^+^ cells) or neighbouring wild‐type cells (Figure 3a'‐a″″, GFP^−^ cells). The nuclei of *Ras^V12^, scrib^−/−^* cells were irregularly shaped (Figure 3a″‐a″″), and we detected chromatin bridge‐like structures (Figure 3a″), micronuclei (Figure 3a″’), and chromatin structures that appeared to be leaking from the nucleus (Figure 3a″″) specifically in *Ras^V12^, scrib^−/−^* clones. These observations suggest that CIN is increased in *Ras^V12^, scrib^−/−^* clones. Note that expression of *Ras^V12^* alone is not sufficient to induce CIN in eye‐antennal discs, indicating that *scrib* loss might be critical for the induction of CIN.

To check if STING signalling was increased in these clones, we measured the mRNA expression of STING targets, such as *Sting‐regulated gene 1* (*Srg1*), *Sting‐regulated gene 2* (*Srg2*), and *Sting‐regulated gene 3* (*Srg3*) (Goto et al. 2018; Holleufer et al. 2021). These targets were unaltered in *Ras^V12^* discs when compared to wild‐type discs (Figure S4). In contrast, these targets were significantly elevated in *Ras^V12^, scrib^−/−^* discs when compared to *Ras^V12^* discs, in a manner dependent on STING (Figure 3b). Together, these results show that cGAS‐STING signalling is elevated specifically in *Ras^V12^, scrib^−/−^* tumours.

### 2.4 Depletion of STING in *Ras^V12^, scrib^−/−^* Tumours Decreases the Biogenesis of Large EVs

We next addressed the role of STING in the production of large EVs. Knockdown of *Sting* in *Ras^V12^, scrib^−/−^* discs using three RNAi lines (*1667R‐1*, *1667R‐3*, and *GD1095*) abolished the induction of the STING target genes (Figure 3b) even though the features associated with CIN persisted regardless of STING depletion (Figure S5). These results further support that the expression of the STING target genes was dependent on STING. Importantly, knockdown of *Sting* in *Ras^V12^, scrib^−/−^* clones also significantly reduced the production of large EVs (Figure 3c,d; Videos S5–S8), indicating that STING is required for large EV biogenesis from *Ras^V12^, scrib^−/−^* tumours. *Sting* knockdown did not reverse the developmental delay caused by *Ras^V12^, scrib^−/−^* tumours. If large EVs are important for inducing the systemic immune response to tumours, STING in *Ras^V12^, scrib^−/−^* tumours should be necessary for the immune response to tumours. Indeed, *Sting* knockdown in *Ras^V12^, scrib^−/−^* tumours impaired the induction of *DptB*, *Drs*, and *Def* in the fat body (Figure 3e,f). These results demonstrate that STING plays an important role in large EV biogenesis and further supports the role of tumour‐derived large EVs in the systemic immune response to *Ras^V12^, scrib^−/−^* tumours.

### 2.5 Activation of cGAS‐STING Signalling Increases the Production of Large EVs From Multiple Human Cancer Cell Lines

**FIGURE 4:**
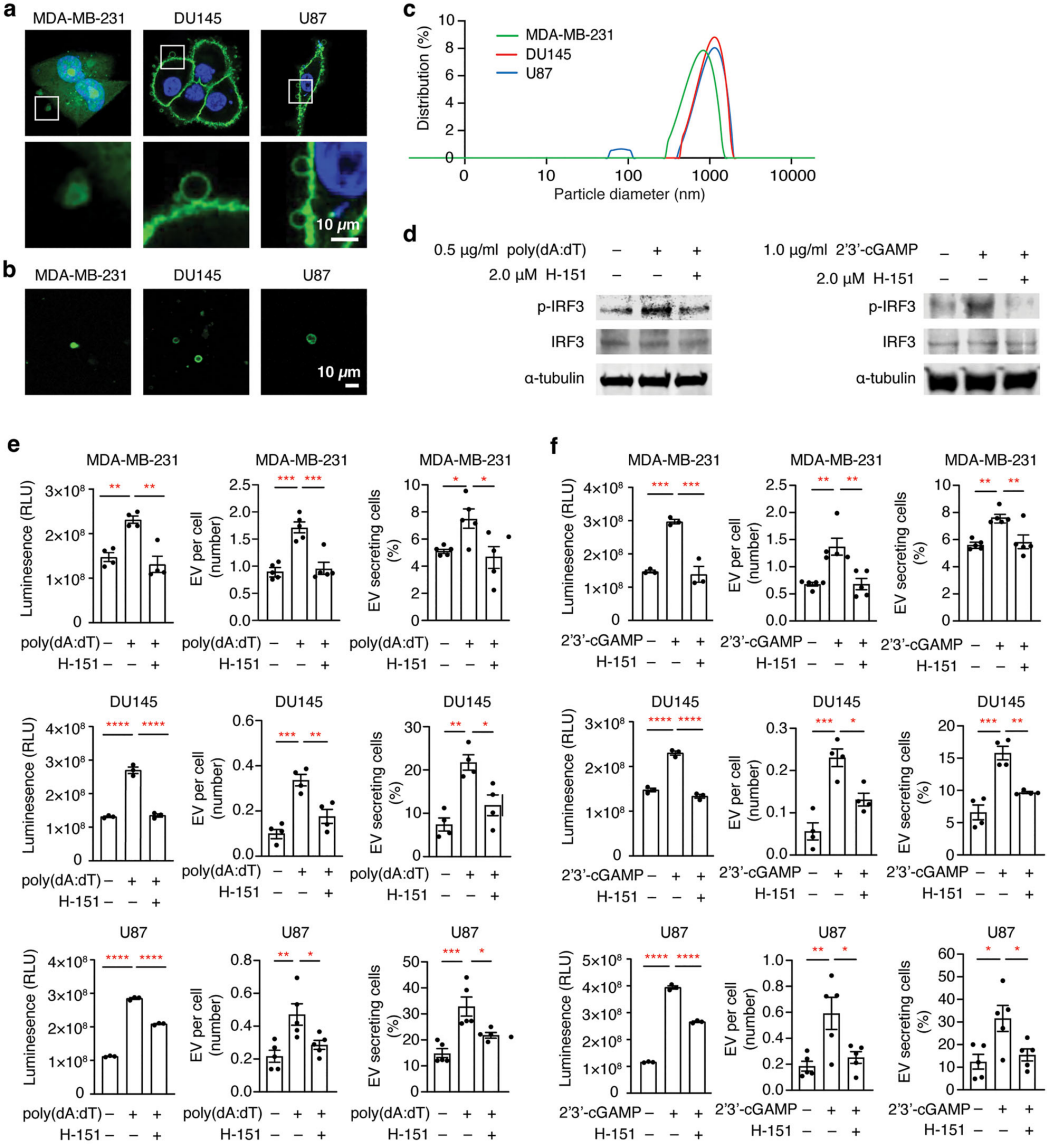
**Activation of cGAS‐STING** signalling **increases the production of large EVs in multiple human cancer cell lines**. (a) Representative images of large EVs from MDA‐MB‐231, U87, and DU145 cells. MDA‐MB‐231 cells express GFP (green). The membrane is labelled with FITC‐CTxB (green) in U87 and DU145 cells, DNA is marked with DAPI (blue). (b) Images of large EVs isolated from MDA‐MB‐231, U87, and DU145 cells. (c) Particle size distribution of isolated EVs from human cancer cells. (d) Western blot of p‐IRF3 to detect STING activity. MDA‐MB‐231 cells were transfected with the indicated concentrations of poly(dA:dT) or 2’3’‐cGAMP, as well as treated with the STING inhibitor H‐151. (e) Quantification of the amount of large EVs produced from cancer cells, the number of large EVs produced per cell, and the percentage of large EV producing cells. Quantifications were performed 24 h after transfection of 0.5 µg/mL poly(dA:dT) with or without 2 µM H‐151 treatment. The leftmost graphs show luminescence‐based quantifications, the middle graphs show large EV counts normalized to cell number, and the rightmost graphs show the percentage of cells producing large EVs. Mean ± SEMs are shown. *N* ≥ 4. *p < 0.05, **p < 0.01, ***p < 0.001, and ****p < 0.0001, one‐way ANOVA. (f) Quantifications of the amount of large EVs produced from cancer cells, the number of large EVs produced per cell, and the percentage of large EV producing cells. Experiments were performed as in e, but cells were transfected with 2’3’‐cGAMP. *N* ≥ 4. *p < 0.05, **p < 0.01, ***p < 0.001, and ****p < 0.0001, one‐way ANOVA.

Given our observations in *Drosophila*, we decided to address whether cGAS‐STING signalling plays a similar role in large EV biogenesis in human cancer cells. It has been shown that the mammary adenocarcinoma cell line MDA‐MB‐231, the prostate carcinoma cell line DU145, and the glioma cell line U87 all produce large EVs (Minciacchi et al. 2015; Morello et al. 2013; Wright et al. 2014). These EVs could be detected adjacent to the cells when they were imaged (Figure 4a). Additionally, we were able to enrich these EVs by adapting the procedures for fractionating large oncosomes (Morello et al. 2013) (Figure 4b,c). The size of the enriched large EVs was ∼1.0 ± 0.68 µm (Figure 4c). We also occasionally detected peaks around 100 nm, which were reminiscent of exosomes (Figure 4c).

Previous studies have shown that transfection of double‐stranded DNA, such as poly(dA:dT), activates the cGAS‐STING pathway (Sun et al. 2013). Additionally, transfection of the cGAS product 2’3’‐cGAMP activates STING, resulting in an increase in phosphorylation of IRF3 (Cheng et al. 2018; Tanaka and Chen 2012). Transfection of poly(dA:dT) or 2’3’‐cGAMP increased phosphorylation of the downstream STING‐effector IRF3 in all three cell lines (Figure 4d). This increase was reversed by the selective small‐molecule inhibitor of STING, H‐151 (Figure 4d). To measure the quantity of EVs produced from these cell lines, we generated a stable cell line constitutively expressing NanoLuc luciferase (NLuc), which is 100‐fold brighter than conventional luciferases (Hall et al. 2012), as a tool to quantify a small amount of EVs by measuring luminescence as a proxy (see Methods). After transfecting poly(dA:dT) or 2’3’‐cGAMP, we assessed EV production by measuring luminescence in large EV‐enriched fractions, and by quantifying EVs detected in culture by imaging. In MDA‐MB‐231, DU145, and U87 cell lines, poly(dA:dT) or 2’3’‐cGAMP transfection significantly increased the quantity of large EVs, and treatment with the STING inhibitor H‐151 reversed this effect (Figure 4e,f). Moreover, transfecting poly(dA:dT) or 2’3’‐cGAMP was sufficient to increase the proportion of large EV producing cells, which was also reversed by treating with H‐151 (Figure 4e,f). These data indicate that activation of STING increases the production of large EVs from human cancer cell lines. Altogether, our results unveil a conserved role of STING in large EV biogenesis from malignant cells.

### 2.6 JNK and FAK Function Downstream of STING to Produce Large EVs

Given that we identified a new role for STING, we sought to uncover the downstream effectors of STING that function in large EV biogenesis. Although the TBK1‐IRF3‐Interferon‐β (IFNβ) axis is characterized as the main effector of STING, IRF3 and interferons are only present in vertebrates (Cai and Imler 2021; Margolis et al. 2017; Patel et al. 2023). Depletion of inhibitor of nuclear factor κB kinase ɛ (IKKɛ), TBK1's closest homolog in *Drosophila*, did not alter large EV production (Figure S6a). Although orthologous effectors of STING, such as IKKɛ, IKKβ, and NF‐κB, exist in *Drosophila*, their depletion in *Ras^V12^, scrib^−/−^* cells had no effect on large EV production either (Figure S6a). Thus, STING may engage a novel signalling axis to induce large EV production.

**FIGURE 5:**
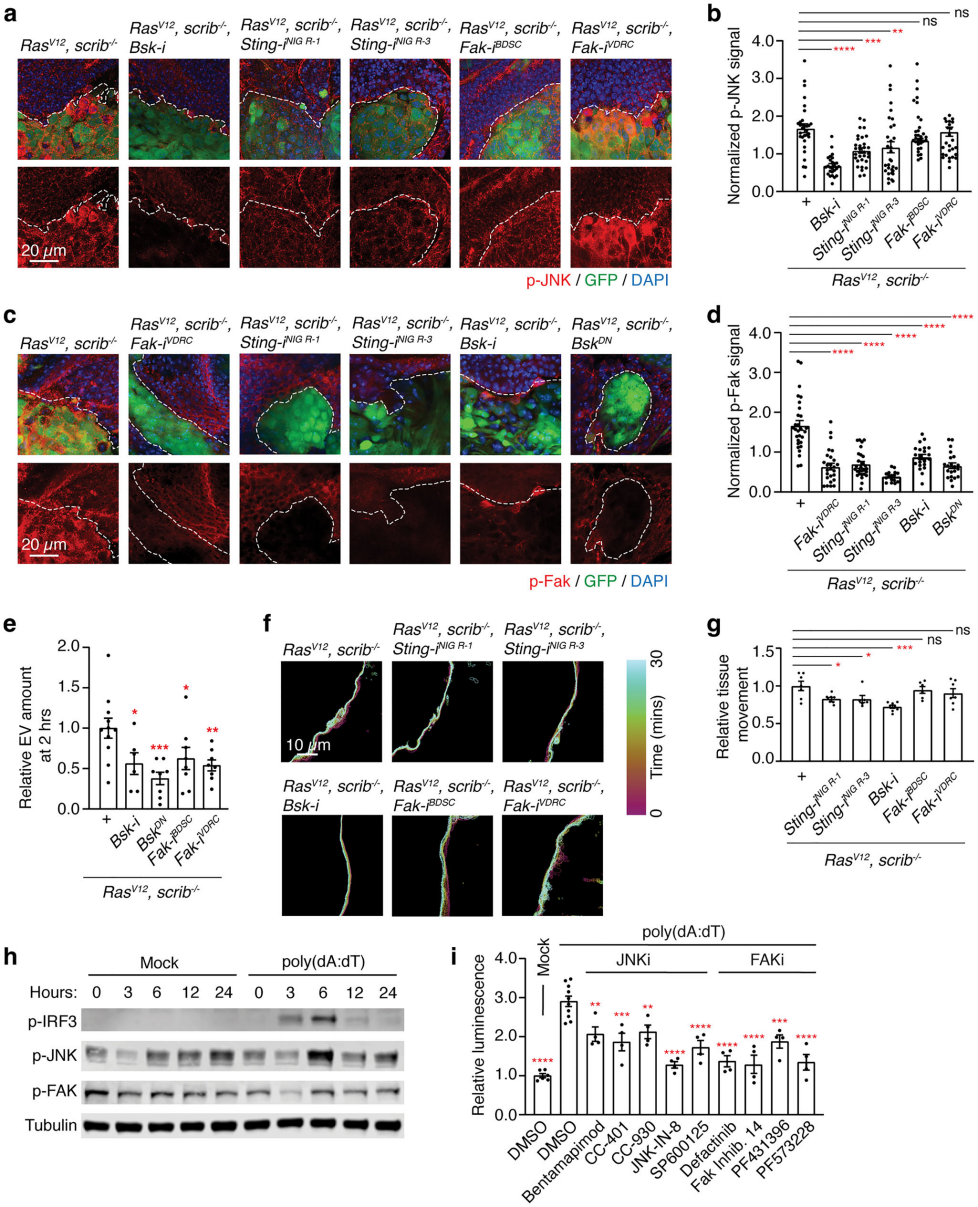
STING is upstream of and acts through JNK and FAK to induce large EV biogenesis. (a) Confocal images of eye discs at 7 d AEL. GFP (green) marks clones with the indicated genotypes; non‐GFP neighbouring cells are wild type. Dotted lines indicate clone boundaries. Anti‐pJNK signals are in shown in red; DAPI (blue) labels nuclei. (b) Quantification of anti‐pJNK signals. pJNK signals in clones (indicated genotypes) are normalized to those in neighbouring non‐clonal cells (wild‐type). *N* ≥ 26. Mean ± SEMs are shown. *p < 0.05, **p < 0.01, ***p < 0.001, and ****p < 0.0001, one‐way ANOVA. (c) Confocal images of eye discs at 7 d AEL. GFP (green) marks clonal cells with the indicated genotypes; anti‐pFAK signals (red); DAPI (blue); dotted lines indicate clone boundaries. (d) Quantification of anti‐pFAK signals. pFAK signals in clones (green) are normalized to those in neighbouring wild‐type non‐clonal cells. *N* ≥ 16. Mean ± SEMs are shown. ****p < 0.0001, one‐way ANOVA. (e) Quantification of GFP^+^ EVs produced from eye discs at 2 h post‐incubation from eye discs from 7 d AEL larvae. Data are normalized to the number of EVs produced by *Ras^V12^, scrib^−/−^* discs. *N* ≥ 6. Mean ± SEMs are shown. *p < 0.05, **p < 0.01, and ***p < 0.001, one‐way ANOVA. (f) Movement of clone perimeters over time. Eye discs were prepared at 7 d AEL for live imaging. Tissue borders were tracked over a 30‐minute time course. Line colours indicate time points, as shown in the legend on the side. (g) Quantification of perimeter movement shown in e. *N* ≥ 6. Mean ± SEMs are shown. *p < 0.05, **p < 0.01, and ***p < 0.001, one‐way ANOVA. See Videos S9–S14. (h) Western blot of MDA‐MB‐231 whole cell lysates prepared at the indicated time points following mock or poly(dA:dT) transfection. (i) Quantification of large EVs derived from NLuc‐expressing MDA‐MB‐231 cells with or without poly(dA:dT) transfection and with or without DMSO (0.1%) or chemical treatment (concentrations indicated in Methods). *N* = 6 (Mock), 10 (DMSO), or 4 (all other samples). Mean ± SEMs are shown. *p < 0.05, **p < 0.01, ***p < 0.001, and ****p < 0.0001, one‐way ANOVA compared to poly(dA:dT)‐transfected and DMSO‐treated measurement.

Previous studies have shown that c‐Jun N‐terminal kinase (JNK) signalling plays an important role in the progression of *Ras^V12^, scrib^−/−^* tumours (Brumby and Richardson 2003; Wu et al. 2010), and we have noticed that *Drosophila* focal adhesion kinase (Fak) phosphorylation is specifically increased in *Ras^V12^, scrib^−/−^* tumours. Thus, we sought to determine how JNK and FAK might interact with STING in *Ras^V12^, scrib^−/−^* tumours. Phospho‐JNK and phospho‐FAK signals were significantly increased in *Ras^V12^, scrib^−/−^* clones compared to neighbouring wild‐type cells (Igaki et al. 2006) (Figure 5a–d). STING depletion in *Ras^V12^, scrib^−/−^* clones significantly decreased both phospho‐JNK and phospho‐FAK signals (Figure 5a‐5d), indicating that STING is required for the activation of JNK and FAK. Importantly, depletion of the JNK ortholog Basket (Bsk), expression of a dominant negative Bsk (Bsk^DN^), or depletion of FAK significantly reduced large EV biogenesis from *Ras^V12^, scrib^−/−^* tumours (Figure 5e), demonstrating that JNK and FAK contribute to large EV biogenesis. We noticed that the edge of *Ras^V12^, scrib^−/−^* clones was highly dynamic (Figure 5f and 5g; Video S9), likely reflecting membrane blebbing (Brassart et al. 2019; Jeppesen et al. 2025; Minciacchi et al. 2015). Interestingly, these edge dynamics were lost when STING or Bsk was depleted in *Ras^V12^, scrib^−/−^* clones while FAK depletion had no effect (Figure 5f and 5g; Videos S10–S14). Thus, JNK and FAK act downstream of STING to drive large EV biogenesis. Moreover, our data suggest that STING engages JNK to control blebbing‐like membrane dynamics. Note that Bsk depletion reduced phospho‐FAK while FAK depletion did not alter phospho‐JNK (Figure 5a‐5d). Thus, JNK might also act on FAK to regulate a process distinct from membrane dynamics for large EV biogenesis.

JNK signalling turns on the apoptotic initiator caspase Dronc in *Ras^V12^, scrib^−/−^* clones while the execution of apoptosis is attenuated due to Ras^V12^ (Igaki et al. 2006; Pagliarini and Xu 2003; Pérez et al. 2017). Given that these EVs also resemble apoptotic bodies in size, we tested the roles of caspases in large EV production. Knockdown of Dronc or the effector caspases (Drice and Dcp‐1) in *Ras^V12^, scrib^−/−^* clones did not alter large EV production (Figure S6b), suggesting that STING signalling leading to large EV biogenesis is distinct from apoptotic signalling.

To determine whether large EV biogenesis correlates with tumour burden, we quantified the volumes of *Ras^V12^, scrib^−/−^* tumours. Although knockdown of either *Sting* or *Fak* decreased large EV production, neither significantly affected tumour volume. Notably, one *Fak* RNAi line resulted in a modest increase in tumour volume. Only the expression of *JNK* RNAi reduced tumour size; however, a substantial tumour mass remained (Figure S7). These results suggest that tumour burden is not directly correlated with large EV biogenesis.

Given the novel role of STING signalling in large EV production, we tested if CIN is sufficient for large EV biogenesis. We knocked down Bub3, a mitotic checkpoint gene, or Peanut (Pnut), a septin, knockdown of which is known to cause aneuploidy, in the context of *Ras^V12^* expression (Gerlach et al. 2018; Morais da Silva et al. 2013). Knockdown of either Bub3 or Pnut with *Ras^V12^* expression generated tumours as previously reported (Gerlach et al. 2018; Morais da Silva et al. 2013). Interestingly, while we observed abnormal nuclear shapes and sizes in both genotypes, we only observed micronuclei with Bub3 knockdown (Figure S8a). Bub3 knockdown increased both large EV production and JNK phosphorylation in the context of *Ras^V12^* expression, while Pnut knockdown had no effect (Figure S8b–d). Thus, the presence of cytosolic DNA and DNA structures like micronuclei correlate with both large EV production and JNK activity. Notably, JNK activation with *Ras^V12^* expression through knockdown of the negative regulator of JNK Puckered (Puc) also generated tumours and increased large EV production (Figure S8b). These findings raise the possibility that cytosolic DNA associated with CIN may be responsible for JNK activation for large EV biogenesis in *Ras^V12^, scrib^−/−^* tumours.

Having identified two kinases downstream of STING that drive large EV biogenesis from *Drosophila* tumours, we investigated whether they play a similar role in human cancer cells. Transfection of poly(dA:dT) increased c‐Jun N‐terminal kinase (JNK) phosphorylation, and focal adhesion kinase (FAK) phosphorylation decreased 3 h post‐transfection but recovered by 6 h (Figure 5h), suggesting that their activity may be controlled by cGAS‐STING signalling. Importantly, inhibition of JNK or FAK caused a significant decrease in poly(dA:dT)‐induced large EV biogenesis (Figure 5i), supporting their importance in STING‐mediated large EV production.

To further interrogate the role of STING in large EV production, we tested the function of TBK1‐IRF3 and IKK‐NFΚB axes in this process. Inhibition of TBK1 or TBK1 and IKKε with poly(dA:dT) transfection had no effect on large EV production (Figure S9a,b). Of the two IKKα/IKKβ inhibitors tested, one caused a decrease in large EV production while the other had no effect (Figure S9c,d). However, IKKα/IKKβ inhibition did not reduce pJNK levels after dsDNA transfection (Figure S9e). Thus, if IKKα/IKKβ play a role in large EV biogenesis, they act independently from the STING‐JNK axis.

Altogether, our observations in *Drosophila* tumours and the MDA‐MB‐231 cell line indicate that STING engages a novel signalling axis comprised of JNK and FAK to drive large EV biogenesis from malignant cells.

### 2.7 Large EVs Activate STING Signalling in Haemocytes, and STING in Haemocytes is Required for the Large EV‐Induced Immune Response

**FIGURE 6:**
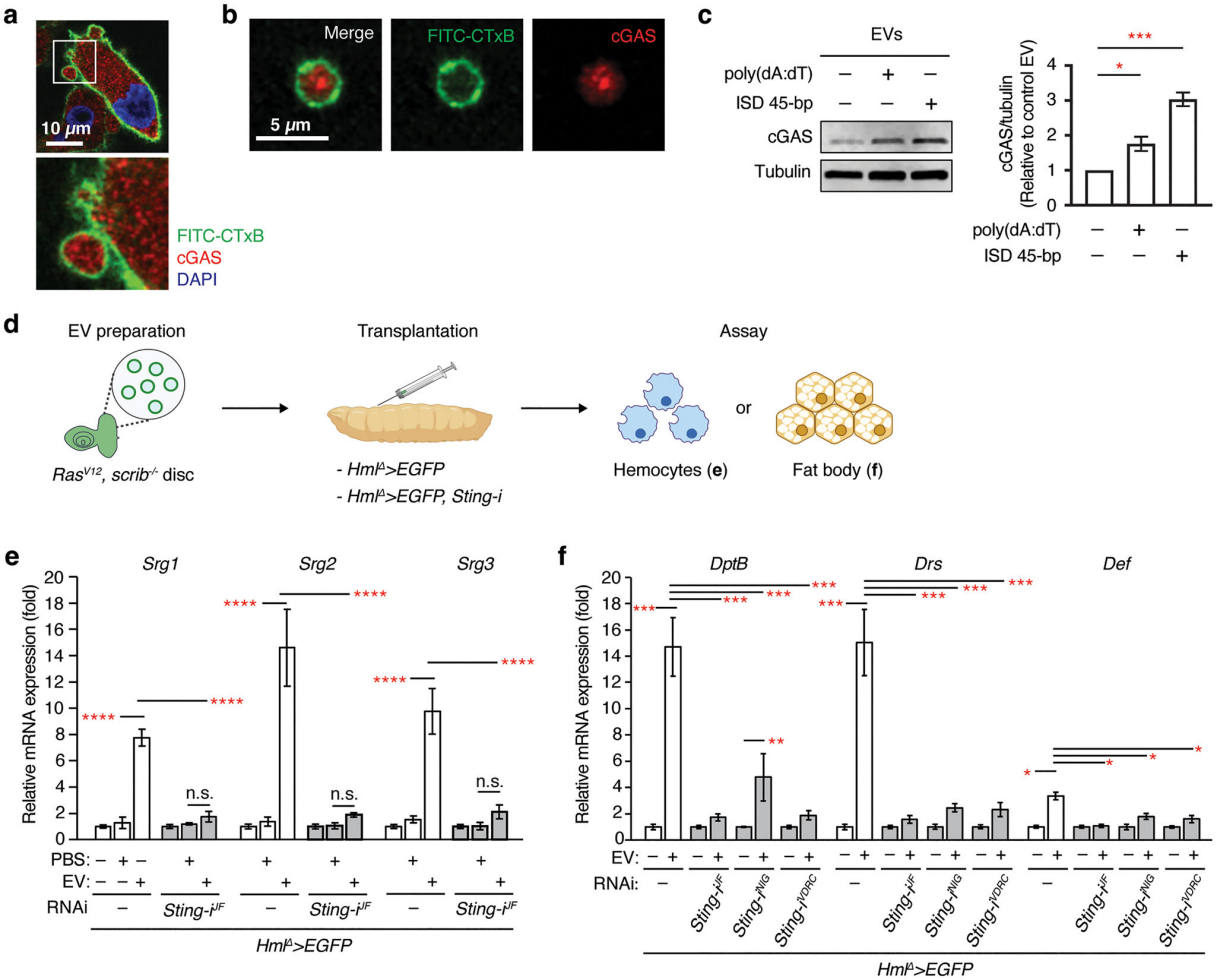
**Sting in haemocytes is necessary for the systemic immune response induced by *Ras^V12^, scrib^−^^/^^−^* disc‐derived large EVs**. (a) Representative images of large EVs budding from MDA‐MB‐231 cells. The plasma membrane is labelled with FITC‐CTxB (green), cGAS signals are shown in red, and DNA is marked with DAPI (blue). (b) Representative images of an isolated large EV. The plasma membrane is labelled with FITC‐CTxB (green) and cGAS signals are shown in red. (c) cGAS detection in large EVs derived from MDA‐MB‐231. Cells were transfected with either 0.5 µg/ml poly(dA:dT) or 5.0 µg/mL 45‐bp ISD. Mean ± SEMs are shown. *N* = 3. *p < 0.05 and ***p < 0.001, one‐way ANOVA. (d) Schematic of large EV injection experiment with haemocyte manipulation. Large EVs derived from *Ras^V12^, scrib^−/−^* discs 8 d AEL were collected and then injected into early 3^rd^ instar larvae (*Hml^∆^>EGFP* or *Hml^∆^>EGFP, Sting‐i*). Haemocytes from injected larvae were collected and subjected to qRT‐PCR to check the expression of STING target genes (e). Fat body was used to check the expression of antimicrobial peptides (f). (e) Relative mRNA expression of STING target genes in haemocytes. Haemocytes isolated from 3^rd^ instar larvae were subjected to qRT‐PCR to check the expression of the STING target genes after injection with PBS or large EVs isolated from *Ras^V12^, scrib^−/−^* eye discs 8 d AEL. Mean ± SEMs are shown. ****p<0.0001, one‐way ANOVA. n.s., not significant. (f) Relative mRNA expression of AMPs in the fat body. Fat body isolated from 3^rd^ instar larvae was subjected to qRT‐PCR to check the expression of antimicrobial peptides after injection with PBS or large EVs isolated from *Ras^V12^, scrib^−/−^* eye discs 8 d AEL. Mean ± SEMs are shown. *p < 0.05, **p < 0.01, ***p < 0.001, one‐way ANOVA.

Since cGAS forms a complex with cytosolic DNA (Du and Chen 2018), it is possible that cGAS might be also loaded into large EVs. Indeed, Clancy et al. showed that cGAS is included in EVs derived from a melanoma cell line (Clancy et al. 2022). We detected cGAS in large EVs derived from MDA‐MB‐231 cells by immunostaining and Western blot (Figure 6a–c). Notably, we found that transfecting with poly(dA:dT) or interferon stimulatory DNA (ISD) significantly increased cGAS levels in large EV fractions (Figure 6c). This result suggests that the activity of cGAS‐STING signalling might be associated with cGAS loading to large EVs, which could be explained by the increased availability of cGAS‐DNA complex in the cytosol.

Considering the role of STING in inducing large EV biogenesis, large EVs generated from *Ras^V12^, scrib^−/−^* discs might also contain cGAS complexed with DNA. Thus, we hypothesized that STING signalling in haemocytes themselves might be required for transducing the signal mediated by large EVs. To test this hypothesis, we first examined whether transplantation of large EV fractions prepared from *Ras^V12^, scrib^−/−^* tumours could increase the expression of STING target genes in haemocytes (Figure 6d). PBS transplantation did not significantly alter the expression of STING target genes in haemocytes (Figure 6e). In contrast, transplantation of large EV fractions prepared from *Ras^V12^, scrib^−/−^* discs significantly increased the mRNA expression of STING target genes in haemocytes (Figure 6e). To test whether haemocyte STING is required for the large EV‐induced increase in STING target gene mRNA levels, we depleted STING specifically in haemocytes by expressing RNAi against *Sting* with the haemocyte driver *Hml^Δ^‐GAL4* (Figure 6d). *Sting* knockdown in haemocytes significantly attenuated the large EV‐induced increase in the expression of STING target gene mRNA (Figure 6e). Taken together, these results demonstrate that large EVs prepared from *Ras^V12^, scrib^−/−^* discs activate STING signalling in haemocytes.

Next, we examined whether STING in haemocytes was required for the EV‐induced systemic immune response. To this end, we transplanted large EV fractions prepared from *Ras^V12^, scrib^−/−^* discs into larvae with depleted STING in haemocytes (Figure 6d,f). As we previously observed, transplantation of large EV fractions from *Ras^V12^, scrib^−/−^* discs increased the mRNA expression of *Drs*, *Dpt*, and *Def* in the fat body (Figure 6f). In contrast, depletion of STING specifically in haemocytes attenuated the induction of the antimicrobial peptides in the fat body (Figure 6f). These results indicate that STING in haemocytes is also required for the systemic immune response induced by large EVs.

## 3 Discussion

**FIGURE 7:**
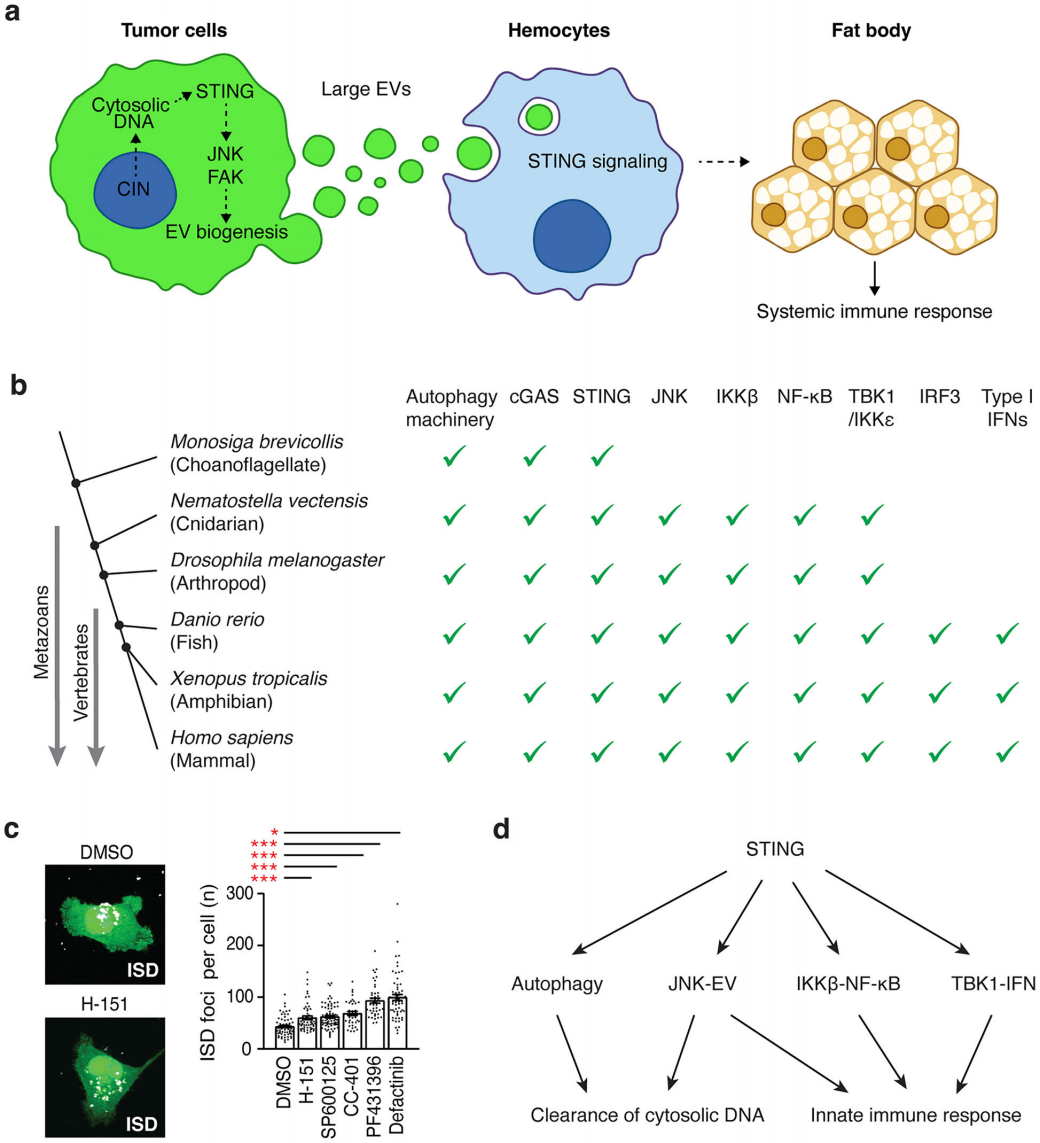
**An expanded view of the axes and functions of STING signalling**. (a) Proposed model of the large EV‐mediated communication between tumours and the immune system. Activation of cGAS‐STING signalling associated with CIN increases the production of large EVs from tumour cells. These large EVs increase STING signalling in haemocytes and induce a systemic immune response to tumours. Thus, STING is necessary in both tumours and immune cells to induce the systemic immune response to tumours, and tumour‐derived large EVs play a key role in the systemic propagation of cytosolic DNA response. (b) An evolutionary tree exploring the conservation of the cGAS‐STING pathway components across species. cGAS, STING, JNK, and autophagy machinery have been identified in essentially all metazoans. JNK is not found in choanoflagellates, the closest living relatives of metazoans. IKKβ‐NF‐κB and TBK1/IKKε appear in cnidarians. IRF3 and type I interferons emerge in vertebrates, including fishes and mammals. (c) Quantification of ISD foci in cells. Cells were transfected with 0.25 µg/mL Cy3‐labelled 100 bp ISD and treated with DMSO (vehicle only), STING inhibitor (5 µM H‐151), JNK inhibitors (10 µM SP600125 and 10 µM CC401), or FAK inhibitors (10 µM PF431396 and 5 µM defactinib). Representative images show cells expressing cytosolic GFP (green), with ISD puncta (grey). Nuclei were stained with DAPI (blue). mean ± SEMs are shown. *N* ≥ 38. *p < 0.05, **p < 0.01, ***p < 0.001, and ****p < 0.0001, one‐way ANOVA. (d) Schematic summarizing the expanded axes and functions of STING signalling. STING engages multiple downstream axes to regulate cytosolic DNA clearance and innate immune responses.

Our study demonstrates that large EV biogenesis from malignant cells is an evolutionarily conserved process. Large EVs derived from *Ras^V12^, scrib^−/−^* tumours are produced by membrane shedding and are larger than exosomes and conventional ectosomes. These EVs resemble large oncosomes and cytoplasts produced from mammalian cancer cell lines (Headley et al. 2016; Minciacchi et al. 2015; Yanez‐Mo et al. 2015). Since these EVs are quite large and labelled with GFP, they are readily traceable in live animals and *ex vivo*. We could detect them inside larvae via live imaging and observe the biogenesis of these EVs from *Ras^V12^, scrib^−/−^* tumours *ex vivo*. We have established a procedure for enriching large EVs produced from *Ras^V12^, scrib^−/−^* discs for further biochemical and functional assays. Interestingly, transplantation of large EVs into wild‐type larvae was sufficient to induce the expression of multiple antimicrobial peptides in the fat body in a manner dependent upon *Drosophila* macrophage‐like cells (Figure 2). Thus, we propose that these EVs are signalling modalities for the communication between tumour cells and the host immune system, which induces a systemic immune response to malignant cells (Figure 7a). Altogether, we have established a novel *in vivo* model allowing us to study large EVs in action by employing the advanced genetic tools available in *Drosophila*. EV‐mediated signalling is a complex process involving multiple steps between donor cells and recipient cells nearby or in different organs. This model provides us an opportunity to closely observe the large EV‐mediated signalling process, from their biogenesis to the physiological consequences of EV signalling in a native context (Figure 7a).

Our study uncovers a role for STING signalling in large EV biogenesis from malignant cells (Figure 7a). Despite the divergence of Arthropoda, including *Drosophila melanogaster*, from Vertebrata ∼700 million years ago (Figure 7b), STING's function in large EV biogenesis from malignant cells is essentially conserved. Notably, STING engages a novel signalling axis comprising JNK and FAK to drive large EV biogenesis from both *Drosophila* and human malignant cells (Figure 5). Although IRF3 and interferons evolved in vertebrates (Cai and Imler 2021; de Oliveira Mann et al. 2019; Margolis et al. 2017; Patel et al. 2023), JNKs can be traced to early metazoans (King et al. 2008) (Figure 7b). EVs have been observed in nearly all model organisms, including some plants and bacteria (Ghanam et al. 2022). Altogether, our study suggests that the STING‐JNK‐EV axis evolved in early metazoans to mount an ancestral immune response against malignant cells prior to the emergence of interferons (Figure 7b). JNKs mediate a wide range of cellular responses to various stresses, including genotoxic stress and infection (Pérez et al. 2017; Ryoo et al. 2004; Diwanji and Bergmann 2019; Wagner and Nebreda 2009; Cui et al. 2007). Positioning JNK downstream of STING may broaden the scope of STING‐mediated response to cytosolic DNA. Thus, it would be interesting to further delineate the STING‐JNK‐EV axis in cancers as well as in various STING‐activating contexts, such as viral infections, and to determine whether additional JNK‐mediated stress responses also constitute part of the STING signalling output (Pérez et al. 2017; Ryoo et al. 2004; Diwanji and Bergmann 2019; Wagner and Nebreda 2009; Cui et al. 2007).

Recent studies have revealed an evolutionarily conserved function of STING in clearing cytosolic DNA through the activation of autophagy (Zhang et al. 2021; Gui et al. 2019). Interestingly, inhibition of STING, JNK, or FAK reduces the clearance of fluorescently labelled dsDNA from MDA‐MB‐231 cells following transfection, which activates STING (Figure 7c). Thus, the STING‐JNK‐EV axis may also contribute to cytosolic DNA clearance by facilitating its export (Figure 7d). Given that STING signalling drives large EV biogenesis, there is a high likelihood that cellular components associated with STING activation—such as cytosolic DNA, cGAS, and 2’3’‐cGAMP—are packaged into EVs. This possibility may explain why tumour‐derived EVs carry the majority of circulating tumour DNA and act as potent modulators of innate immunity (Headley et al. 2016; Clancy et al. 2022; Wortzel et al. 2024; Tsering et al. 2024; Vagner et al. 2018; Li et al. 2021; Zhang et al. 2015; Tkach et al. 2022).

Our *Drosophila* model illustrates how STING signalling propagates from malignant cells to immune cells via large EV biogenesis to induce a systemic immune response to malignant cells (Figure 7a). Multiple features associated with CIN are manifested in *Ras^V12^, scrib^−/−^* tumours, leading to the activation of STING signalling (Figure 3). Since STING is activated by cytosolic DNA, an increase in large EV biogenesis could raise the chance for cytosolic DNA to be included in these EVs. Stimulation of cGAS‐STING signalling by transfecting double‐stranded DNA also increases the cGAS protein levels detected in large EVs (Figure 6c). Interestingly, recent studies have shown that EVs derived from tumour cells can activate STING signalling in recipient cells (Tkach et al. 2022; Allen et al. 2022). Notably, transplantation of the *Ras^V12^, scrib^−/−^* tumour‐derived large EV fractions is sufficient to activate cGAS‐STING signalling in haemocytes. Finally, we demonstrate that STING in haemocytes is required to induce the EV‐mediated humoral immune response (Figure 6e,f). Altogether, our study provides insights into how STING signalling in tumours propagates to immune cells to induce a systemic immune response to tumours in an animal.

Immune responses in the *Ras^V12^, scrib^−/−^* tumour model likely intersect. Toll signalling is elevated in these tumours and it increases JNK activity (Mishra‐Gorur et al. 2019). Therefore, STING and Toll signalling may converge on JNK activation, subsequently promoting tumour progression and large EV biogenesis. In addition, haemocytes recruited to tumours by reactive oxygen species (ROS) and PDGF‐ and VEGF‐related factor 1 (Pvf1) secrete the Toll ligand Spätzle (Spz) (Pastor‐Pareja et al. 2008; Zhao et al. 2025). Toll activation leads to a positive feedback loop through increased expression of Spz and expression of AMPs including Drosomycin and Defensin in the larval fat body (Shia et al. 2009). Given the elevation of these AMPs in the *Ras^V12^, scrib^−/−^* tumour model, it is likely that Toll is activated as well as Immune deficiency (IMD). The mechanism of this crosstalk should be further explored as no interaction between STING and Toll signalling has been reported (Martin et al. 2018).

In humans, it is likely that large EVs interact with tumour associated macrophages or circulating monocytes (Popena et al. 2018; Tang et al. 2024). First, like many tumour‐derived EVs, MDA‐MB‐231 EVs contain chromosomal DNA alone or in complex with cGAS (Clancy et al. 2022; Kim et al. 2024). We hypothesize that uptake of these EVs by macrophages could activate STING signalling, leading to release of interferons and pro‐inflammatory cytokines. In addition, Heat Shock Protein 70 (HSP70) may be present on the surface of MDA‐MB‐231 EVs (Jordan et al. 2020). HSP70 binds to the Toll‐like receptors TLR2 and TLR4 on the surface of macrophages, leading to NF‐ΚB activation (Linder and Pogge von Strandmann 2021). Thus, these large EVs may have parallel immune‐activating effects in *Drosophila* and human systems.

In summary, our study consolidates that large EVs derived from malignant cells are bona fide signalling entities and establishes *Drosophila* as a suitable model for studying these EVs in action. We discover the conserved role of STING signalling in driving large EV biogenesis from malignant cells, which is important for the propagation of STING signalling from tumour cells to immune cells to induce a systemic immune response. EVs produced from cancer cells can play both anti‐ and pro‐tumour roles depending on the context (Choi et al. 2017; Minciacchi et al. 2017; Marar et al. 2021). Thus, manipulation of STING signalling could be a way to increase or decrease large EV production to achieve a desired outcome for cancer therapy. Importantly, future studies utilizing *Drosophila* genetics will allow us to answer the fundamental questions underlying the large EV‐mediated signalling process. In particular, the *Drosophila* model could be useful for understanding how large EVs are processed in immune cells to induce systemic immune response and what physiological consequences they elicit, which might allow the discovery of additional targets for manipulating EV‐mediated responses.

## 4 Materials and Methods

### 4.1 Fly Husbandry and Genetics

Flies are reared in standard molasses‐agar medium at room temperature. Dr. Tian Xu (Yale University) kindly provided the following lines: 1) *yw;; FRT82B*, 2) *w; UAS‐Ras^V12^; FRT82B*, 3) *w; UAS‐Ras^V12^; FRT82B, scrib^1^/TM6B*, and 4) *yw, ey‐Flp1; act>y^+^>GAL4, UAS‐GFP.S65T; FRT82B, tub‐GAL80* (Pagliarini and Xu 2003). Dr. Andreas Bergmann (UMass Chan Medical School) kindly provided *Sco/CyO, tub‐RFP; UAS‐Ras^V12^, FRT82B, scrib^1^/TM6B* ^71^. All other lines were obtained from the Bloomington Drosophila Stock Centre (BDSC), the Vienna Drosophila Resource Centre (VDRC) or NIG‐Fly. All detailed information for fly stocks is summarized in Table S1. Larvae were age‐controlled within 24 h. The parental generation was transferred into new vials, then transferred out after a 24‐h egg‐laying window. We define days AEL as days after parents are initially flipped into vials. Larvae were grown at 24°C.

### 4.2 Confocal Time‐Lapse Imaging

The previously described procedures (Lee et al. 2020) were modified for *ex vivo* live imaging of eye discs. *Ex vivo* live‐imaging medium comprises Schneider's *Drosophila* medium (Thermo Fisher Scientific, 21720024), 2% FBS (Life Technologies, 16140071), and 0.5% penicillin‐streptomycin (Thermo Fisher Scientific, 15140122). Eye imaginal discs from wandering 3rd instar larvae (6‐day AEL for *Ras^V12^* or 7‐ or 8‐day AEL for *Ras^V12^*, *scrib^−/−^* and other genotypes) were dissected in imaging media and then mounted on a 35 mm glass bottom dish (MatTek, P35G‐1.0‐14‐C). To prevent squeezing of the discs, four dots of vacuum grease were applied as square corners on the glass bottom dish, approximately 5 mm apart. One hundred microlitres of imaging media was added to the centre of the dish, and then the dissected discs were placed in the centre. A round cover glass was gently placed on top of the vacuum grease and approximately 1 mL of imaging medium was added to the dish to prevent dehydration and facilitate gas exchange. Then, the dish lid was put in place. The samples were imaged from the bottom side. Time‐lapse imaging of the whole mount larva was prepared in a similar way.

Serial z‐stack images of discs were obtained for 2 h using a Leica SP8 laser scanning confocal microscope with 20× dry or 40× water immersion objectives, then were processed into z‐projection videos with the Leica LAS X software. The number and diameter of the GFP‐positive vesicles detected outside the discs were quantified using NIH ImageJ software by counting EVs moving in‐frame at the 2 h mark.

### 4.3 Clonal Tissue Movement Imaging and Quantification

Images of eye disc clone edges were taken over a 30‐min period using the 20× dry objective of a Leica SP8 spinning confocal microscope. Tissues were dissected and prepared similarly as for *ex vivo* live imaging. Image analysis was performed using NIH ImageJ software (Schindelin et al. 2012;Rueden et al. 2017). Drift correction was performed, and clone edges were skeletonized. Individual pixels were tracked using TrackMate (Ershov et al. 2022). The pixel distance travelled between time points was calculated and averaged for each imaginal disc using Python on the Visual Studio Code interface.

### 4.4 Collection of EVs From Drosophila Eye Discs

Eye antennal discs from 20 to 30 late 3rd instar larvae were dissected in *ex vivo* live‐imaging medium. Dissected discs were incubated in 200 µL *ex vivo* live‐imaging medium for 4 h at room temperature and subjected to differential centrifugations to enrich large EVs. First, the tube containing eye discs and media was centrifuged at 500 × *g* for 5 min to remove the eye discs and cells. The supernatant was then centrifuged at 3000 × *g* for 10 min to obtain a pellet containing large EVs. The supernatant was centrifuged at 3000 × *g* for 10 min again to recover residual large EVs. The pellets were merged and resuspended in 100 µL PBS. The resuspended sample was centrifuged at 3000 × *g* for 10 min to pellet large EVs. The pellet was resuspended in 10–20 µL PBS for subsequent experiments.

For injection control experiments, small EVs (sEVs) and large EV washes were also utilized. For this experiment, 50–60 late 3rd instar larvae were dissected. To isolate sEVs, the supernatant was reserved after the first 3000 × *g* spin so that cell debris and large EVs were removed. This supernatant was centrifuged at 100,000 × *g* for 30 min to collect sEVs. The sEV pellet was resuspended in 30 µL PBS for experiments. To collect the large EV wash, large EVs were resuspended in 30 µL of PBS after the second 3000 × *g* spin. An additional 10‐min, 3000 × *g* spin was performed. The 30 µL of PBS supernatant was removed as the large EV wash fraction, and the large EV pellet was resuspended in 30 µL of PBS for injection.

### 4.5 EM

Eye discs were dissected and fixed overnight in 0.1 M sodium cacodylate buffer (pH 7.2) supplemented with 4% glutaraldehyde. The samples were washed, postfixed in 1% osmium tetroxide for 90 min, rinsed, stained in 1% uranyl acetate, dehydrated in ethanol solutions, and embedded in epoxy resin (Epon Araldite). Serial sections (80 nm) were aligned and viewed on a JEOL‐1230 transmission electron microscope with an AMT XR80 camera.

### 4.6 Collection of Circulating Haemocytes

Late 3rd instar larvae were vortexed in PBS with glass beads for 5 min to detach the sessile haemocytes. Individual larvae were placed in each well of a PTFE‐printed multi‐test slide glass (Electron Microscopy Sciences, #63424‐06), bled in 10 µL of ice‐cold PBS, and then subjected to standard immunocytochemistry protocols.

### 4.7 Immunofluorescence of Fly Tissues

Tissues dissected in PBS were fixed in 4% paraformaldehyde (Electron Microscopy Sciences) for 20 min then washed three times for 5 min each time with PBST (PBS supplemented with 0.2% Triton X‐100) for permeabilization. Tissue samples were then incubated in blocking buffer (PBST supplemented with 5% normal goat serum) for 1 h at room temperature. Then, tissue samples were incubated with primary antibody in blocking buffer overnight at 4°C. Primary antibodies included anti‐pJNK (1:200, Cell Signalling, 4668), anti‐pFak (1:200, Cell Signalling, 700255), and anti‐lamin (1:1000, DSHB, ADL67.10). The tissue samples were washed three times with PBST for 5 min each time and then were incubated with secondary antibody for 2–3 h at room temperature. Stained tissues were washed 3 times with PBST and mounted with Vectashield (Vector Laboratories, Cat# H‐1000). Fluorescence micrographs were acquired with Leica SP8 laser scanning confocal microscope with 40x/1.25 objective lens. Fiji software was used for further adjustment and assembly of the acquired images.

### 4.8 Microinjection of EVs in *Drosophila* Larvae

Early 3rd instar larvae with appropriate genotypes were removed from food, washed in PBS two times, and then rinsed in 70% ethanol for sterilization. Thin‐walled glass filaments (World Precision Instruments, tw100f) were pulled into injection needles and backfilled with 0.5 µL of EV fractions in PBS. The needle was assembled to the microinjector (World Precision Instruments, PV820 Pneumatic PicoPump), and larvae were injected by handheld motion by targeting the space between the dorsal trunks. The injected droplet ranged from 30 to 50 µm in diameter. After injection, the larvae were recovered in normal food for 18 h at room temperature and then subjected to subsequent experiments.

### 4.9 Quantitative Real‐Time PCR

Total RNA from eye discs, fat body, or isolated haemocytes was isolated with TRIzol (Invitrogen, Cat# 15596026). RNA (1 µg) was used to produce cDNA with iScript Reverse Transcription Supermix (Bio‐Rad, Cat#1725120). The cDNA was subjected to quantitative real‐time PCR with iTaq Universal SYBR Green Supermix (Bio‐Rad, Cat#1708840) and CFX‐96 (Bio‐Rad). RpL32 was used for normalization. The fold change in RNA expression compared to the control was calculated and plotted for relative mRNA expression. Primers used for RT‐qPCR are described in Table S1.

### 4.10 *Drosophila* Tumour Volume Quantification

Z‐stacks of tumours were taken using a Leica SP8 laser scanning confocal microscope with the 20× dry objective with a 1.5 µm step size. In Fiji, a threshold was set for the GFP channel for each z‐stack, and the GFP^+^ area was calculated for each slice in µm. This area was summed and multiplied by 1.5 to approximate total tumour volume.

### 4.11 Cell Culture

The human prostate carcinoma (DU145), human glioblastoma (U87), and human breast adenocarcinoma (MDA‐MB‐231) cell lines were purchased from ATCC. GFP‐expressing MDA‐MB‐231 cells were generated by lentiviral transduction (Sigma‐Aldrich, ORFGFPV). Cells were cultured in DMEM (Life Technologies, 11‐965‐118) supplemented with 10% FBS (Life Technologies, 16140‐071) and 1% penicillin‐streptomycin (Life Technologies, 15‐140‐122) and were regularly tested for mycoplasma (Applied Biological Materials, G328). 0.5 µg/mL poly(dA:dT) (Invivogen, tlrl‐patn‐1), 1.0 µg/mL 2’3’‐cGAMP (ApexBio, B8362), or 5.0 µg/mL 45‐bp ISD was transfected into 3.0 × 10^6^ cells using Lipofectamine 3000 (Invitrogen, L3000) according to the manufacturer's instruction. Transfected cells were incubated for 24 h before analysis.

The GFP‐expressing or NanoLuc‐expressing MDA‐MB‐231 cell lines were generated by lentiviral transduction (Sigma–Aldrich, ORFGFPV). Briefly, HEK392T cells in DMEM were transfected with pR8.91, VSV‐G and either pLenti‐CMV:Flag‐NLuc‐SV40:PuroR or pLenti‐CMV:GFP‐SV40:PuroR plasmids using Lipofectamine 3000. pLenti‐CMV:Flag‐NLuc‐SV40:PuroR was obtained from Addgene (#183045; http://n2t.net/addgene:183045; RRID: Addgene_183045). Media was collected after 48 h, diluted to various concentrations, and a final concentration of 8 µg/mL polybrene was added. MDA‐MB‐231 cells were reverse transduced. After 48 h, the media was exchanged for complete media with 5 µg/mL puromycin. Complete media with puromycin was changed every 23 days for 10 days to complete selection.

### 4.12 Preparation of 45‐bp Interferon Stimulatory DNA (45‐bp ISD)

Oligonucleotides (TACAGATCTACTAGTGATCTATGACTGATCTGTACATGATCTACA) labelled with Cy5 and their complementary oligonucleotides were ordered from Integrated DNA Technologies and then annealed to prepare 45‐bp interferon stimulatory DNA (45‐bp ISD). Equal amounts of the oligonucleotides were dissolved in 10 mM Tris, pH 7.4, 50 mM NaCl, heated up to 90°C, and then decreased the temperature to 15°C by 1°C per minute.

### 4.13 Immunofluorescence of Human Cell Lines

Cells were cultured on glass bottom dish (MatTeK, P35G‐1.5‐20‐C) at 37°C in humidified air with 5% CO_2_. After transfection with poly(dA:dT), 2’3’‐cGAMP, or 45‐bp ISD, DU145 and U87 cells were labelled with 1.0 µg/mL FITC‐conjugated cholera toxin B subunit (CTxB) (Sigma‐Aldrich, C1655) in DMEM for 10 min on ice and then fixed with 4% paraformaldehyde in PBS for 15 min at room temperature. Cells were permeabilized with 0.01% Triton X‐100 in PBS for 15 min at room temperature and incubated with a blocking solution (1% BSA in PBS) for 60 min at room temperature. Cells were stained with anti‐cGAS antibody (1:1000; Cell signalling Technology, 15102) overnight at 4°C and then stained with Alexa 594 antibodies (1:1000; Invitrogen, A11012) for 1 h at room temperature. After washing cells in PBS, cells were stained with 1 mg/mL DAPI. Images were acquired using a Leica SP8 confocal microscope with 40× oil or 63× oil objective lenses.

### 4.14 Isolation of EVs From Human Cancer Cell Lines

Culture media was replaced with DMEM without FBS after washing twice with DMEM. After incubating cells in the DMEM without FBS for 24 h, we collected the DMEM and then centrifuged at 2000 × *g* for 20 min at 4°C to remove large debris and floating cells. To remove any residual cells and large debris, we filtered the supernatants through 5.0 µm PES syringe filter. The filtrates were centrifuged at 8000 × *g* for 40 min at 4°C to precipitate EVs. The pellets were washed by adding in cold PBS or cold DMEM and then centrifuging at 8000 × *g* for 40 min at 4°C.

For quantification of EV amount, the samples were incubated with 1.0 µg/mL FITC‐CTxB in DMEM for 10 min on the ice. The samples were centrifuged at 8000 × *g* for 30 min at 4°C to collect EVs. The pellets were resuspended in cold PBS and then centrifuged again at 8000 × *g* for 30 min at 4°C to wash out free FITC‐CTxB. The collected pellets were suspended in PBS. The fluorescence intensity of FITC‐CTxB was quantified using SpectraMax i3 microplate reader (Molecular Devices). For the MDA‐MB‐231 cell line stably expressing GFP, the GFP fluorescence was directly measured from the collected EV samples.

We used a Litesizer 500 (Anton Paar) to analyse particle size distribution. Collected EV samples were diluted in PBS if necessary and analysed at a scattering angle of 175° at 15°C.

To quantify the effects of JNK, FAK, and other inhibitors in tissue culture, Nluc‐expressing MDA‐MB‐231 cells were used. After passaging, 2.2 × 10^5^ cells were seeded in each well of 12‐well plates. 20–24 hours later cells were transfected as described. Transfected cells were treated with 0.1% DMSO or the following chemicals 3 h post‐transfection: PF573228 (5 µM), PF431396 (5 µM), Defectinib (5 µM), PND1186 (0.5 µM), FAK Inhibitor 14 (5 µM), SP600125 (10 µM), CC‐401 (5 µM), CC‐930 (5 µM), Bentamapimod (5 µM), JNK‐IN‐8 (5 µM), GSK8612 (1 or 10 µM), BAY‐985 (1 or 10 µM), GSK, BMS‐345541 (1 or 10 µM), or BAY 11–7082 (0.5 or 5 µM). EVs were isolated and quantified 18 h later. Prior to EV isolation, cells were observed for detachment, cell death, or alterations in growth to ensure cell number or death did not significantly affect experiment outputs (data not shown).

To isolate EVs from 12‐well plates, supernatant was filtered using 5.0 µm 20 mm PES syringe filters (Tisch Scientific, SPEC18113), and EVs were isolated as described above using DMEM for washes and final resuspension. All spins were done in 1.7 mL Axygen microcentrifuge tubes (MCT‐175). To quantify EV amount, the resuspended EVs were transferred to an opaque white 96‐well plate with one well between samples to reduce cross talk. Nano‐Glo Luciferase Assay System (Promega, N1110) was used as directed to detect luminescence. Luminescence was measured using a SpectraMax i3x (Molecular Devices).

### 4.15 Immunofluorescence of EVs

The isolated EVs were fixed with 1% paraformaldehyde in PBS for 5 min. After fixation, EVs were permeabilized with 0.001% Triton X‐100 in PBS for 5 min on ice. EVs were recovered by centrifuging at 8000 × *g* for 30 min and then washed by resuspending in cold PBS and centrifuging at 8000 × *g* for 30 min. The recovered EVs were resuspended in PBS with anti‐cGAS antibody (1:500; Cell signalling Technology, 15102) and incubated for 2 h at 4°C with gentle rotation. The EVs were recovered by centrifuging at 8000 × *g* for 30 min at 4°C. The pellet was resuspended in PBS with Alexa Fluor 594 antibody (1:500, Invitrogen, A11012) and then incubated for 1 h at 4°C. EVs were incubated with 1 µg/mL FITC‐CTxB in PBS for 10 min on the ice. The mixture was centrifuged at 8000 × *g* for 30 min to recover EVs. The recovered EVs were washed in cold PBS. The pellets were dissolved in Vecta Shield mounting medium (Vector Laboratories, H‐1000‐10) and mounted. The images were acquired using a Leica SP8 laser scanning confocal microscope with 63X oil objective lenses.

### 4.16 Immunoblotting

The cells were lysed in RIPA buffer containing 50 mM Tris‐HCl (pH 7.4), 0.1% SDS, 0.5% sodium deoxycholate, 150 mM NaCl, 1% NP40, and fresh Halt Protease and Phosphatase Inhibitor Cocktail (Thermo Fisher Scientific, 78442) on ice for 20 min. Lysates were collected and centrifuged at 12,000 rpm for 20 min to remove debris. Protein concentration was determined using Thermo Scientific Pierce BCA protein assay kit. The samples were prepared in 4X Laemmli sample buffer (Bio‐Rad, 1610747) supplemented with 355 mM 2‐mercaptoethanol and were heated at 80°C for 10 min. 30–60 µg protein samples were electrophoresed in 8%–16% Mini‐PROTEAN TGX Stain‐Free Protein Gels (Bio‐Rad, 456–1103) and then transferred onto PVDF membranes (Millipore, IPVH00010) using Mini Trans‐Blot cell (Bio‐Rad, 1703930). The membrane was blocked with 5% non‐fat dry milk (Bio‐Rad, 1706404XTU) in TBS‐T (0.1% Tween 20) for 45 min at room temperature. For phospho‐antibodies, Intercept Protein‐Free Blocking Buffer (LICORbio, 927–80001) was used for blocking. The membrane was probed with anti‐cGAS antibody (1:2000; Cell signalling Technology, 15102), anti‐phospho‐IRF3 antibody (1:1000; Cell signalling Technology, 29047), mouse‐anti‐IRF3 antibody (1:1000; DSHB, PCRP‐IRF3‐1D11), anti‐pJNK antibody (1:1000; Cell signalling Technology, 9255), anti‐pFAK antibody (1:1000; Cell signalling Technology, 8556) and/or mouse‐anti‐alpha‐tubulin antibody (1:5000: Fisher Scientific, 14‐4502‐82). The blots were incubated with IRDye 800CW goat anti‐rabbit IgG antibody (LI‐COR Biosciences, 926–32211) or IRDye 800CW goat anti‐mouse IgG antibody (LI‐COR Biosciences, 926–32210). The signals were visualized using the LI‐COR Odyssey CLx Western blot scanner.

## 5 Statistical Analysis

All statistical analyses were performed using GraphPad Prism 9 and Microsoft Excel. The statistically significant difference was calculated by one‐way ANOVA. *p* values below 0.05 were considered statistically significant. Sample sizes were chosen empirically based on the observed effects.

## Author Contributions

**Jiae Lee**: conceptualization, methodology, visualization, writing – original draft, investigation, validation, formal analysis, writing – review and editing. **Annabel Vernon**: conceptualization, visualization, methodology, writing – original draft, investigation, validation, formal analysis, writing – review and editing. **Hyung Joon Park**: conceptualization, methodology, visualization, writing – original draft, validation, investigation, formal analysis. **Young V. Kwon**: conceptualization, methodology, visualization, writing – original draft, writing – review and editing, project administration, funding acquisition, resources, supervision.

## Conflicts of Interest

The authors declare no competing interest.

## Supporting information




**Supporting Information**: jev270289‐sup‐0001‐SuppMat.docx

**Supporting Information**: jev270289‐sup‐0011‐video1.mp4

**Supporting Information**: jev270289‐sup‐0012‐video2.mp4

**Supporting Information**: jev270289‐sup‐0013‐video3.mp4

**Supporting Information**: jev270289‐sup‐0014‐video4.mp4

**Supporting Information**: jev270289‐sup‐0015‐video5.mp4

**Supporting Information**: jev270289‐sup‐0016‐video6.mp4

**Supporting Information**: jev270289‐sup‐0017‐video7.mp4

**Supporting Information**: jev270289‐sup‐0018‐video8.mp4

**Supporting Information**: jev270289‐sup‐0019‐video9.mp4

**Supporting Information**: jev270289‐sup‐0020‐video10.mp4

**Supporting Information**: jev270289‐sup‐0021‐video11.mp4

**Supporting Information**: jev270289‐sup‐0022‐video12.mp4

**Supporting Information**: jev270289‐sup‐0023‐video13.mp4

**Supporting Information**: jev270289‐sup‐0024‐video14.mp4

## Data Availability

All study data are included in the article and/or Supplementary Information.
