# Supplementary material for: Systemic Propagation of STING Signalling via Generation of Large Extracellular Vesicles
Source: J Extracell Vesicles. 2026 May 9;15(5):e70289. doi: 10.1002/jev2.70289 (PMC13157586; doi:10.1002/jev2.70289)
Supplement: Supplementary file 1 — Supporting Information: jev270289‐sup‐0001‐SuppMat.docx [file JEV2-15-e70289-s008.docx]

Supplementary information for

**Systemic Propagation of STING Signaling Via Generation of Large Extracellular Vesicles**

Jiae Lee^1,2^†, Annabel Vernon^1^†, Hyung Joon Park^1, ‡^†, and Young V. Kwon^1^*

**Affiliations:**

^1^Department of Biochemistry, University of Washington; Seattle, WA 98195, USA.

^2^Department of Biological Sciences, California State University Long Beach; Long Beach, CA 90815, USA.

^‡^ Present address: Department of Oral Pathology, School of Dentistry, Chonnam National University; Gwangju, South Korea

†These authors contributed equally to this work.

*Corresponding author. Email: [ykwon7@uw.edu](mailto:ykwon7@uw.edu)

**This PDF file includes:**

Supplementary Figures 1 to 9

Supplementary Video Legends 1 to 14

Supplementary Table 1

**
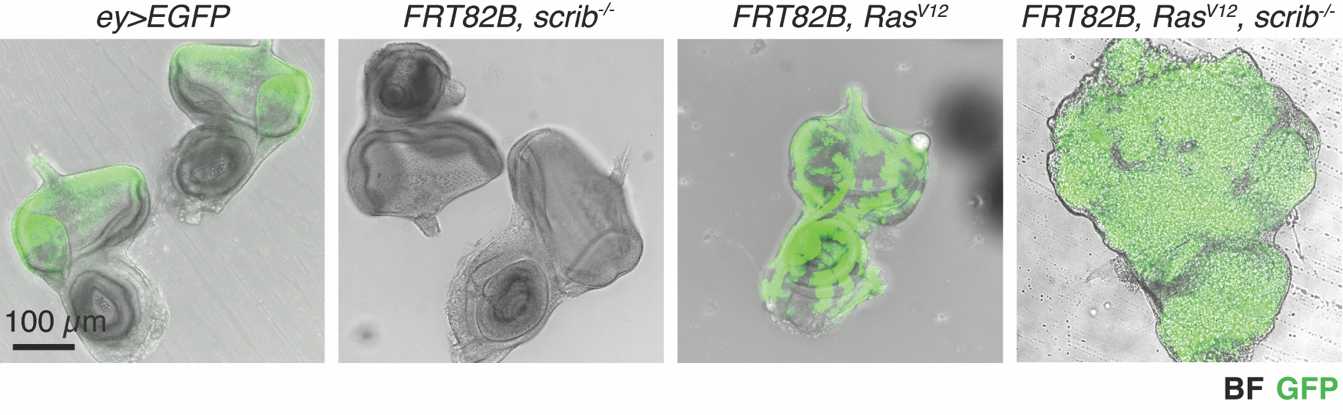
**

**Supplementary Figure 1. Images of eye–antennal discs.** In the first panel, GFP is expressed using the eye disc-specific driver *eyeless*-GAL4 (*ey*>*eGFP*). In the remaining panels, mitotic clones were generated in eye discs using mosaic analysis with a repressible cell marker (MARCM). Eye-antennal discs were dissected from larvae 8 d (*FRT82B* or *Ras^V12^, scrib^-/-^)* or 6d (all other genotypes) AEL. The genotypes of GFP^+^ mitotic clones are indicated. BF, brightfield

**
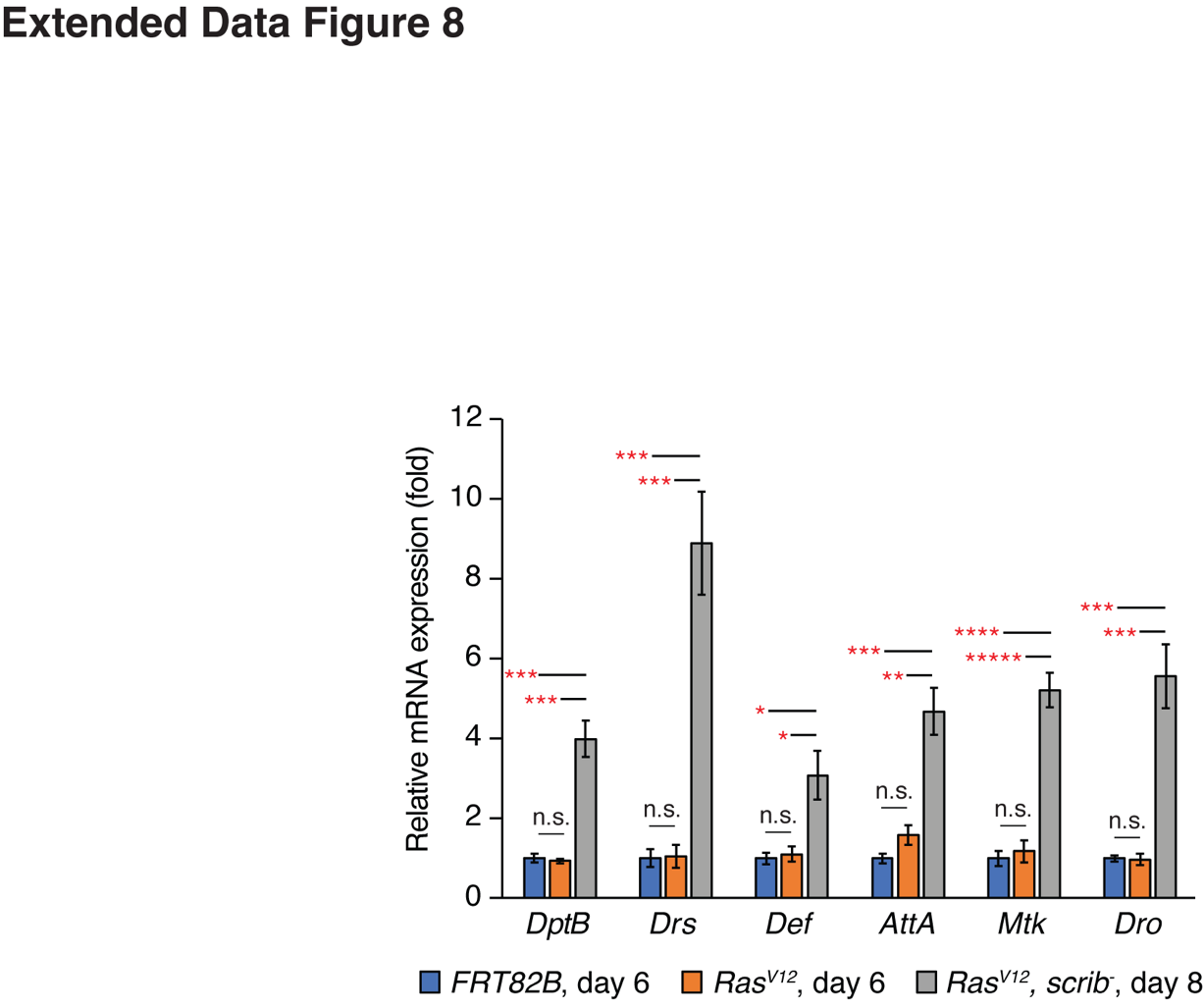
**

**Supplementary Figure 2. Expression of antimicrobial peptides in the fat body is specifically elevated in larvae bearing *Ras^V12^, scrib^-/-^* tumors.** Quantification of antimicrobial peptide transcripts from the fat body from day 6 d or 8 d AEL larvae with eye discs expressing MARCM clones of *FRT82B* (wild-type control), *Ras^V12^*, or *Ras^V12^, scrib^-/-^* were subjected to RT-qPCR. Mean ± SEMs are shown. **p* < 0.05, ***p*<0.01, ****p*<0.001, *****p*<0.0001, n.s.=not significant by one-way ANOVA.

**
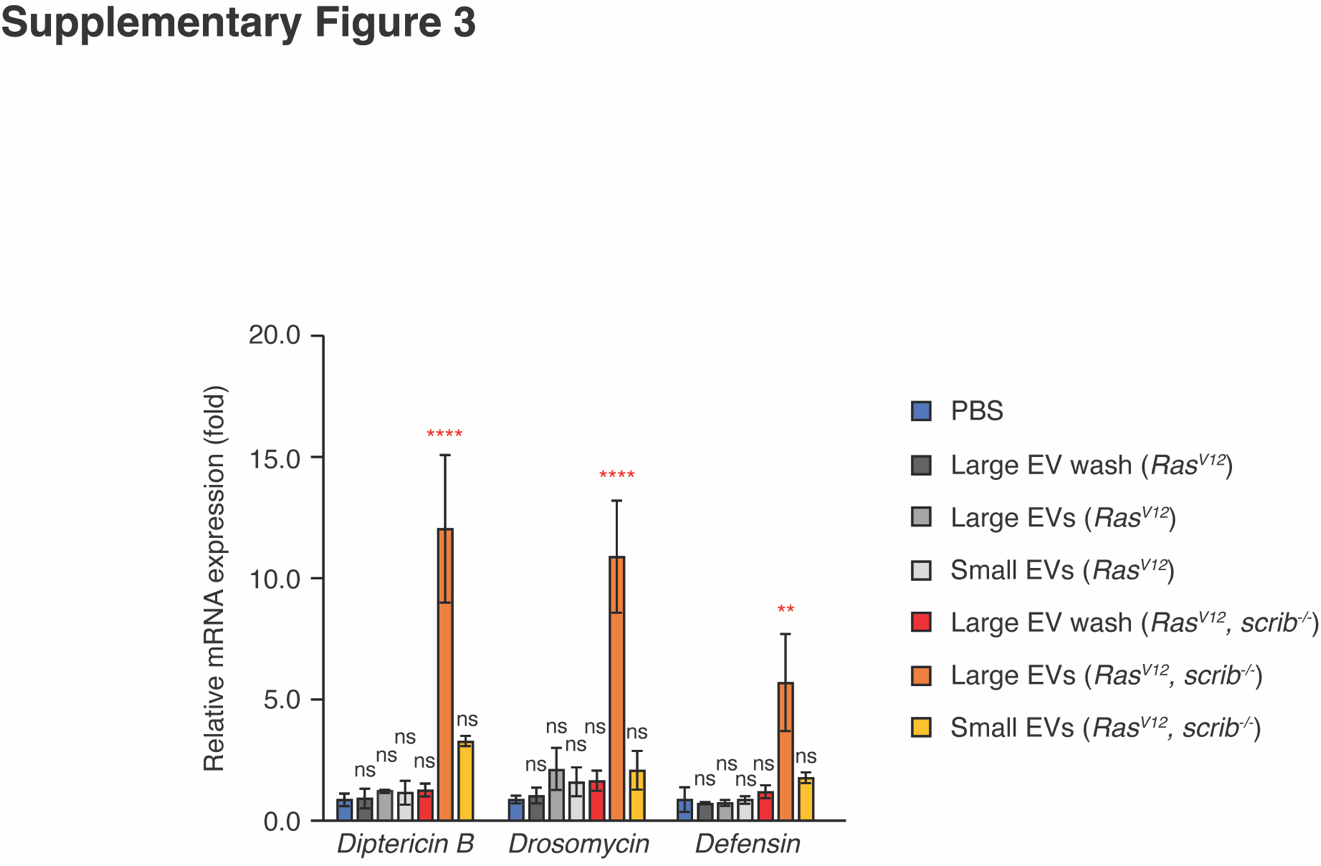
**

**Supplementary Figure 3. Induction of AMP expression is specific to large EVs.** Expression of AMPs in the fat body from 3^rd^ instar larvae 24 hours after injection with PBS, large EVs, the wash fraction of large EV preparation, or small EVs from *Ras^V12^* (6 d AEL) or *Ras^V12^, scrib^-/-^* (8 d AEL) eye discs. Mean±SEMs are shown. ***p*<0.01, *****p*<0.0001, one-way ANOVA. The experiment was repeated 2 times with similar results.

**
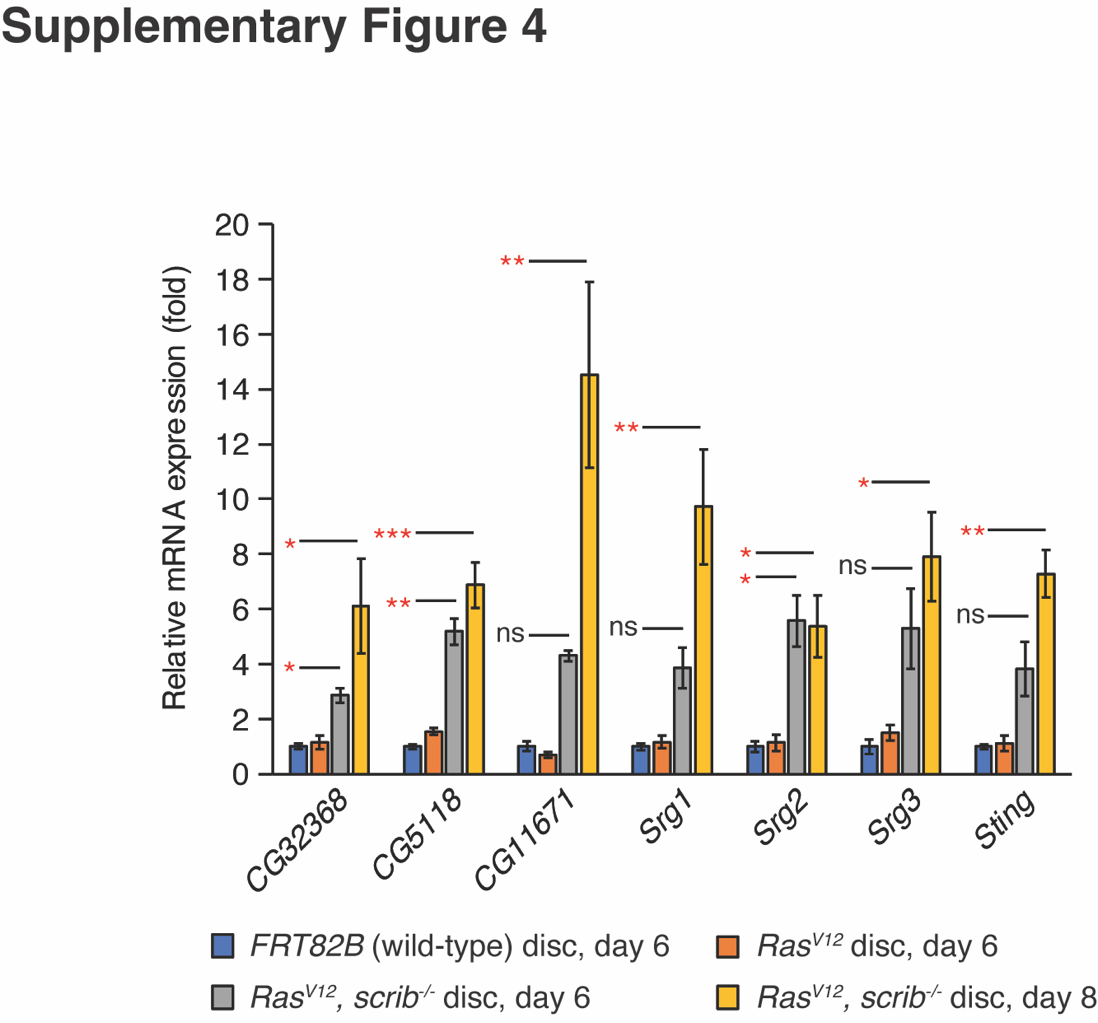
**

**Supplementary Figure 4. STING signaling is elevated in *Ras^V12^, scrib^-/-^* eye disc tumors.** Expression of STING target genes in eye discs from 6 d AEL larvae harboring *FRT82B* (wild-type control) or *Ras^V12^*, and 8 d AEL larvae harboring *Ras^V12^, scrib^-/-^* clones measured by RT-qPCR. Mean±SEMs are shown. **p*<0.05, ***p*<0.01, ****p*<0.001, one-way ANOVA. The experiment was repeated 3 times with similar results.

**
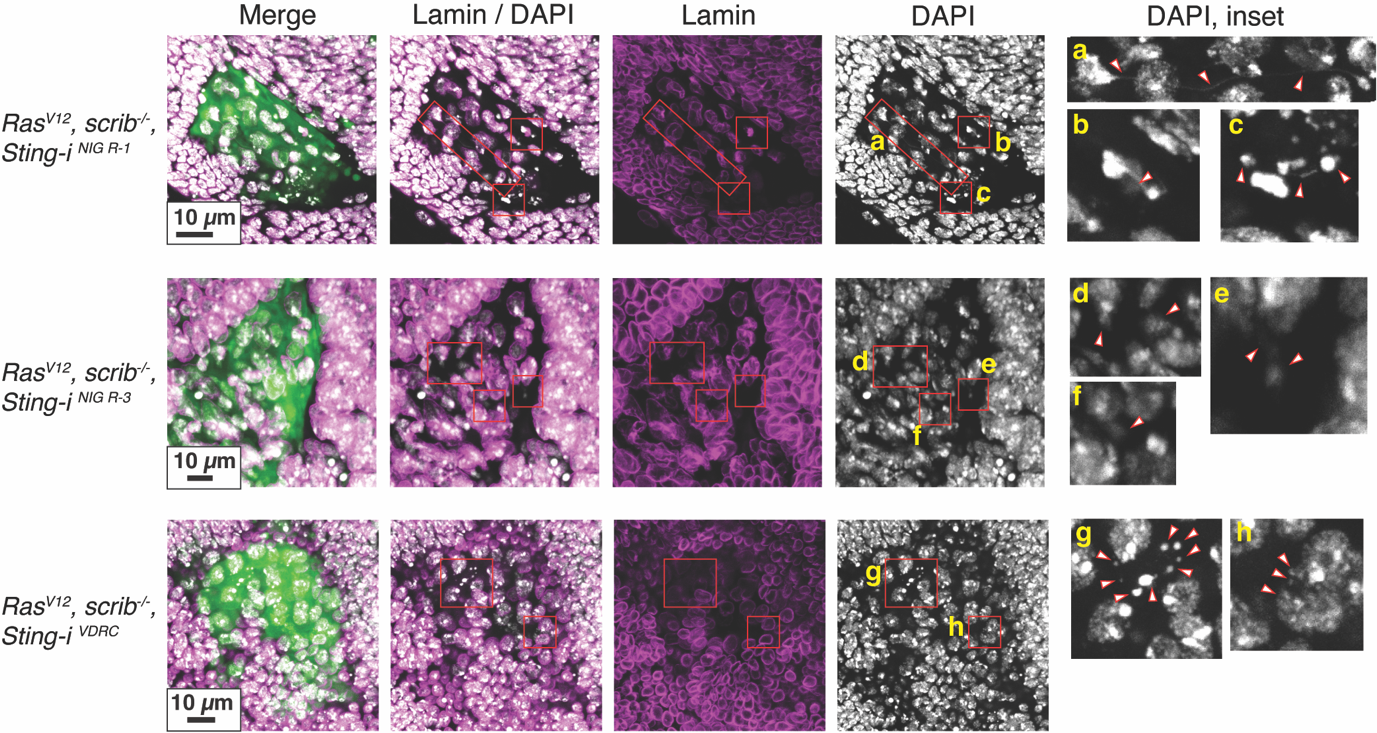
**

**Supplementary Figure. 5. Features associated with chromosomal instability (CIN) persist in *Sting* RNAi-expressing *Ras^V12^, scrib^-/-^* clones.** Confocal images of eye discs containing *Ras^V12^, scrib^-/-^*, *Sting-i* clones (green) from 8 d AEL larvae. Insets and arrowheads highlight features associated with CIN and cytosolic chromatin. Clones are marked by GFP (green), the nuclear envelope is labeled with anti-Lamin staining (magenta), and DNA is stained with DAPI (grey).

**
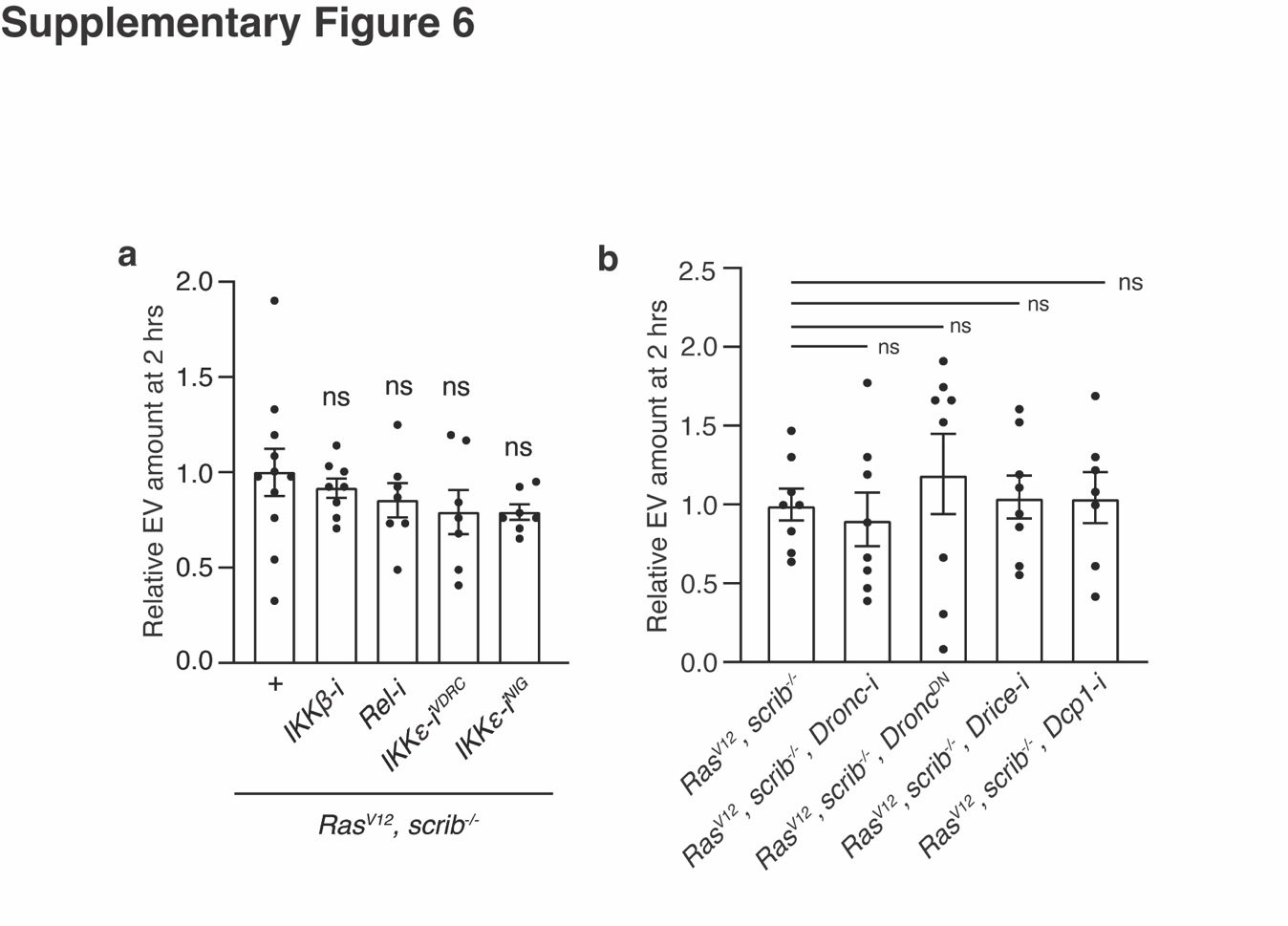
**

**Supplementary Figure 6. EV production is independent of canonical STING signaling and caspase activity. a.** Quantification of EVs from *Ras^V12^, scrib*^-/-^ tumors with knockdown of the indicated enzyme in the cGAS-STING pathway. Mean ± SEMs are shown. ns = not significant, one-way ANOVA, N≥7. **b.** Quantification of EVs produced by *Ras^V12^, scrib*^-/-^ tumors. *Death regulator Nedd2-like caspase3* RNAi (*Dronc-i*), *Death related ICE-like caspase* RNAi (*Drice-i*), *Death caspase-1* RNAi (*Dcp-1-i*), or dominant negative *Dronc* (*Dronc^DN^*) was expressed in *Ras^V12^, scrib*^-/-^ eye disc tumors. Eye disc clone genotypes are indicated. All discs are from larvae 7 d AEL. N≥7. Mean ± SEMs are shown. ns = not significant, one-way ANOVA,

**
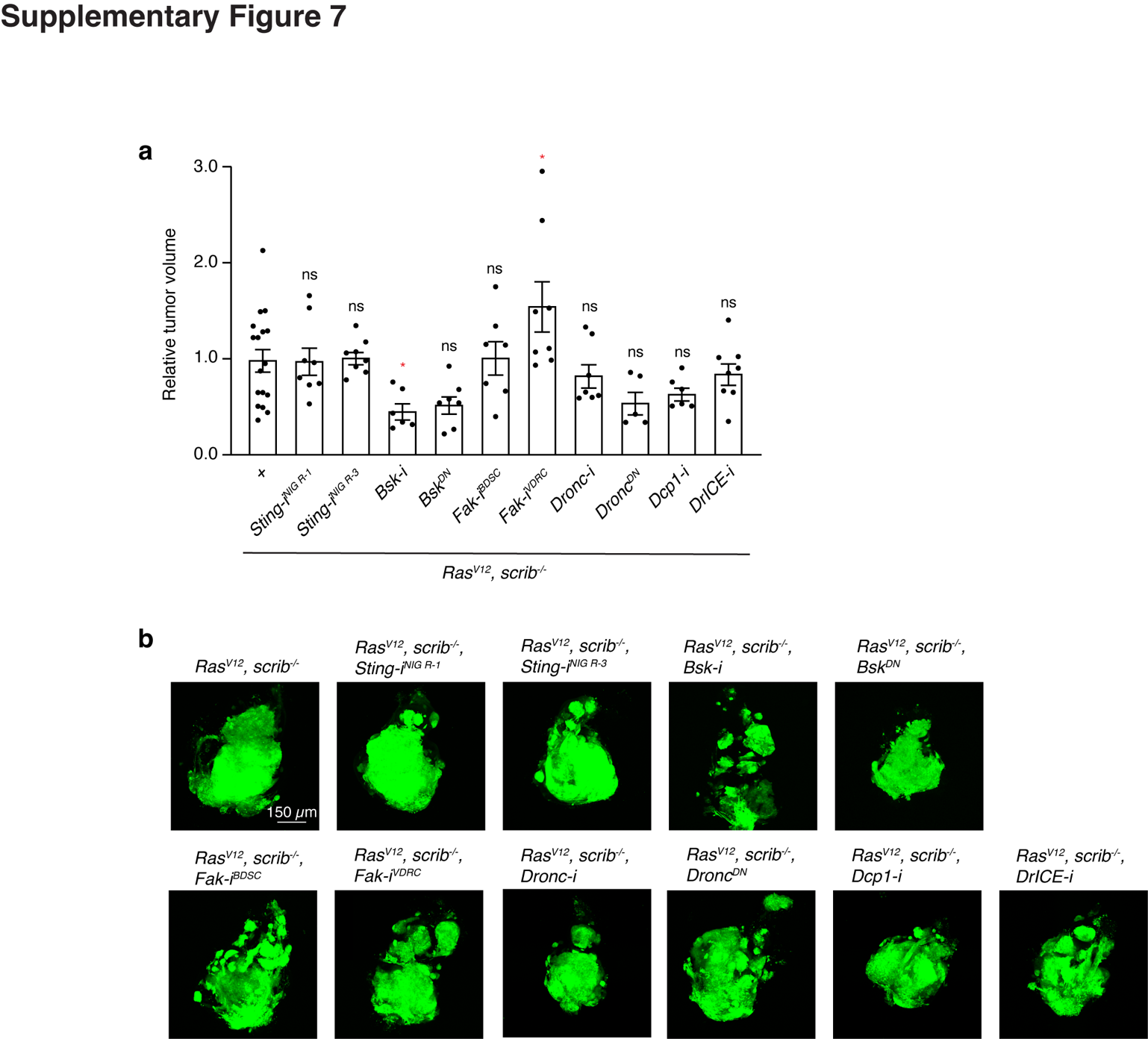
**

**Supplementary Figure 7. Tumor volume effects of RNAi lines. a.** Quantification of GFP^+^ tumor volume of *Ras^V12^, scrib^-/-^* or *Ras^V12^, scrib^-/-^* with the indicated RNAi or dominant negative (DN) proteins. Values are normalized to *Ras^V12^, scrib^-/-^* tumor volume. All larvae were 7 d AEL. See Methods for quantification details. N≥6. Mean±SEMs are shown. **p*<0.05, one-way ANOVA. **b.** Representative maximum z-projections of *Ras^V12^, scrib^-/-^* tumors or *Ras^V12^, scrib^-/-^* tumors with the indicated RNAi or DN.

­­
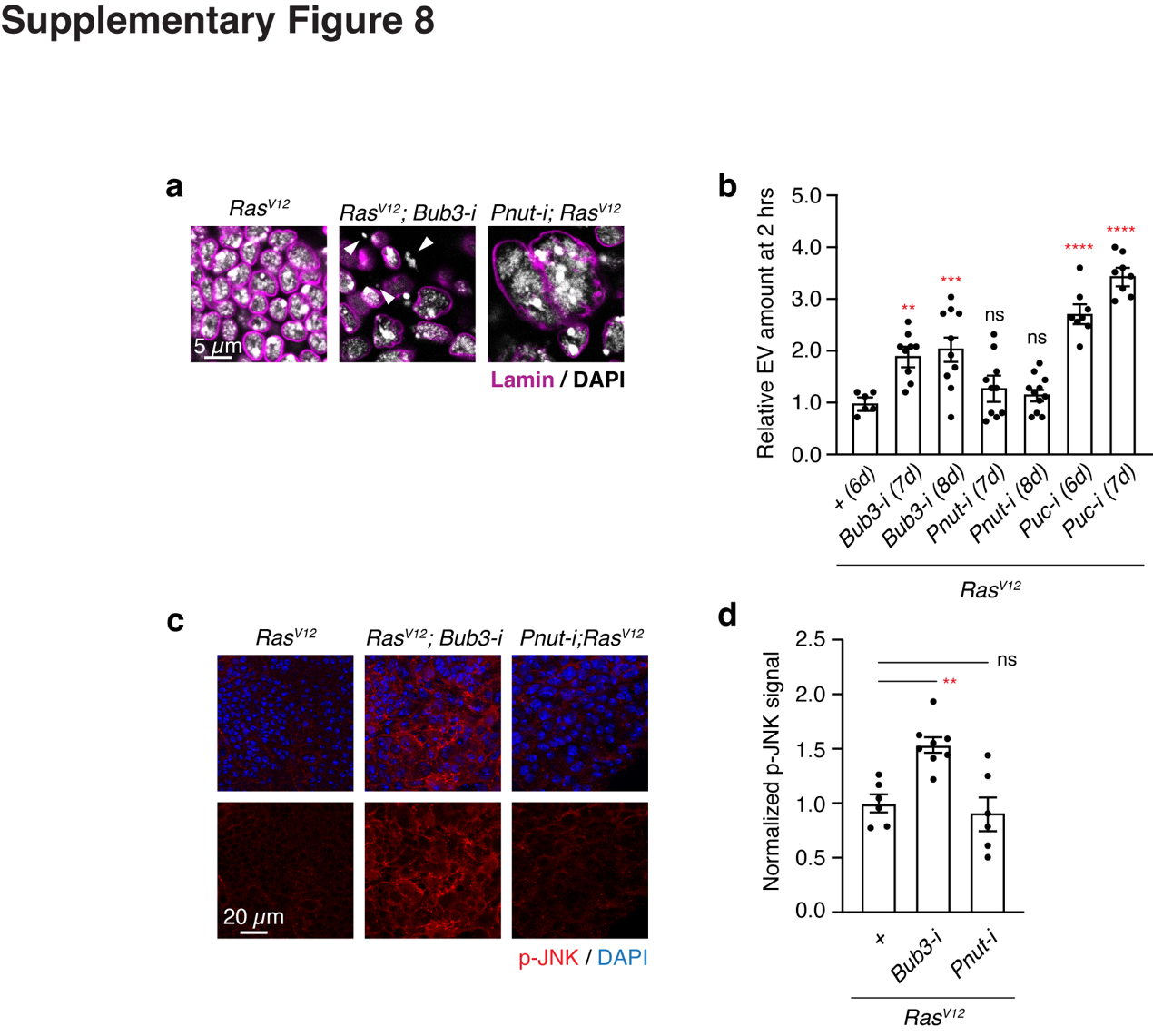


**Supplementary Figure 8. Chromosomal instability and JNK activation may be sufficient to drive large EV production in the context of *Ras^V12^.* a.** Confocal images of *Ras^V12^, Ras^V12^; Bub3-i, or Ras^V12^; Pnut-i* tumor nuclei from eye discs 7 d AEL. Arrowheads indicate micronuclei, which are associated with chromosomal instability. The nuclear envelope is stained with anti-Lamin antibody (magenta) and DNA is stained with DAPI (grey). **b.** Quantification of GFP^+^ EVs produced from tumors of the indicated genotypes at 2 hours post-incubation. Data are normalized to the number of EVs produced by *Ras^V12^* discs. Larval age (days AEL) is indicated for each genotype. N≥6. Mean±SEMs are shown. ***p*<0.01, ****p*<0.001, and *****p*<0.0001. **c and d.** Representative confocal images and quantifications of mean p-JNK signal intensity in the tumors of the indicated genotypes. *Ras^V12^* larvae were 6 d AEL, and other genotypes were 7 d AEL. Anti-pJNK signals are shown in red; DAPI (blue) labels nuclei. N≥6. Mean±SEMs are shown. ***p*<0.01, one way ANOVA.


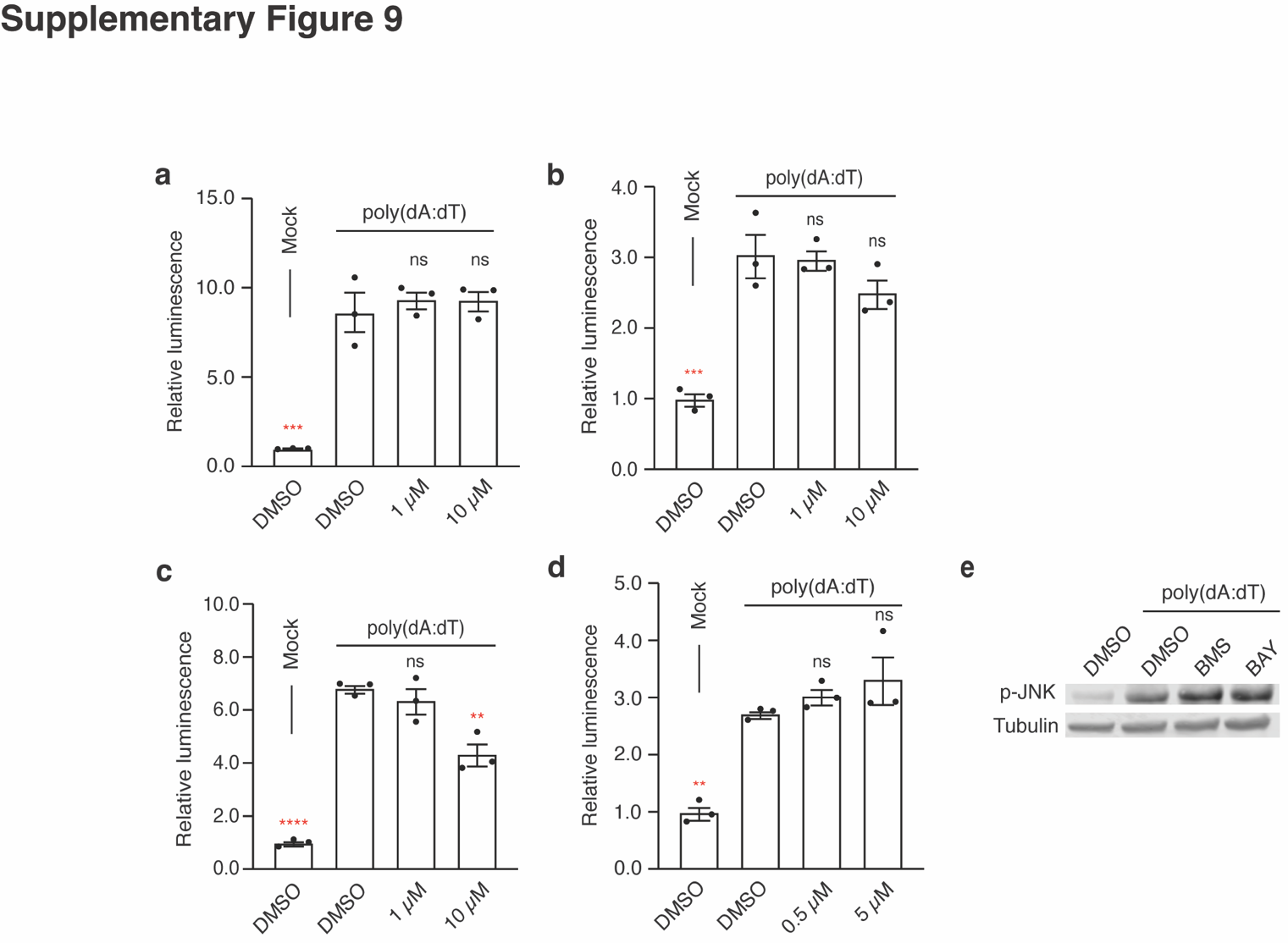


**Supplementary Figure 9. IKKα/β may contribute to large EV biogenesis downstream of STING in MDA-MB-231 cells. a-d.** Quantification of large EVs derived from NLuc-expressing MDA-MB-231 cells with or without 0.5 µg/mL poly(dA:dT) transfection and with DMSO (0.1%) or chemical treatment (**a.** TBK1 inhibitor GSK8612, **b.** TBK1 and IKKε inhibitor BAY-985, **c.** IKKα/β inhibitor BMS-345541, **d.** IKKα/β inhibitor BAY 11-7082) at the indicated concentrations. N = 3, Mean±SEMs are shown. ***p*<0.01, ****p*<0.001, ****p*<0.0001, one-way ANOVA. **e.** Western blot of MDA-MB-231 whole cell lysates prepared at 6 hours after mock or poly(dA:dT) transfection and treatment with DMSO (0.1%), BMS-345541 (10 µM), or BAY 11-7082 (5 µM).

**Supplementary Video Legends**

**Supplementary Video 1.** Whole mount live imaging of the anterior region of a larva with *Ras^V12^, scrib^-/-^* clones in the eye-antennal discs at 10-day AEL (z-projection of 33.3 µm stack, duration: 30 min, 3 fps). Anterior to the upper right.

**Supplementary Video 2.** Whole mount live imaging of the anterior region of a larva with *Ras^V12^* clones in the eye-antennal discs at 6-day AEL (z-projection of 33.3 µm stack, duration: 30 min, 3 fps). Anterior to the right.

**Supplementary Video 3.** Ex vivo live imaging of *Ras^V12^* clones in eye disc (z-projection of 10 µm stack, duration: 15 min, 3 fps).

**Supplementary Video 4.** Ex vivo live imaging of *Ras^V12^, scrib^-/-^* clones in eye disc (z-projection of 10 µm stack, duration: 15 min, 3 fps).

**Supplementary Video 5.** Ex vivo live imaging of *Ras^V12^, scrib^-/-^* clones in eye disc (z-projection of 10 µm stack, duration: 22 min, 3 fps).

**Supplementary Video 6.** Ex vivo live imaging of *Ras^V12^, scrib^-/-^, Sting-i^NIG R-1^* clone in eye disc (z-projection of 10 µm stack, duration: 22 min, 3 fps).

**Supplementary Video 7.** Ex vivo live imaging of *Ras^V12^, scrib^-/-^, Sting-i^NIG R-3^*  clone in eye discs (z-projection of 10 µm stack, duration: 22 min, 3 fps).

**Supplementary Video 8.** Ex vivo live imaging of *Ras^V12^, scrib^-/-^, Sting-i^VDRC^* clone in eye disc (z-projection of 10 µm stack, duration: 22 min, 3 fps).

**Supplementary Video 9.** Ex vivo live imaging of the edge of a tumor from a *Ras^V12^, scrib^-/-^* larva 7 days AEL (z-projection, duration: 30 mins, 7 fps).

**Supplementary Video 10.** Ex vivo live imaging of the edge of a *Ras^V12^, scrib^-/-^, Sting-i^NIG R-1^* eye disc clone 7 days AEL (z-projection, duration: 30 mins, 7 fps).

**Supplementary Video 11.** Ex vivo live imaging of the edge of a *Ras^V12^, scrib^-/-^, Sting-i^NIG R-3^* eye disc clone 7 days AEL (z-projection, duration: 30 mins, 7 fps).

**Supplementary Video 12.** Ex vivo live imaging of the edge of a *Ras^V12^, scrib^-/-^, Bsk-i* eye disc clone 7 days AEL (z-projection, duration: 30 mins, 7 fps).

**Supplementary Video 13.** Ex vivo live imaging of the edge of a *Ras^V12^, scrib^-/-^, Fak-i^BDSC^* eye disc clone 7 days AEL (z-projection, duration: 30 mins, 7 fps).

**Supplementary Video 14.** Ex vivo live imaging of the edge of a *Ras^V12^, scrib^-/-^, Fak-i^VDRC^* eye disc clone 7 days AEL (z-projection, duration: 30 mins, 7 fps).

**Supplementary Table 1. List of reagents**

| **REAGENT or RESOURCE** | **SOURCE** | **IDENTIFIER** |
| --- | --- | --- |
| Antibodies | | |
| Anti-Peroxidasin (pxn) (Rabbit polyclonal) | Gift from Dr. Jiwon Shim (Hanyang Univ.) | https://doi.org/10.14348/molcells.2017.0287 |
| Anti-Lamin (Mouse monoclonal) | Developmental Studies Hybridoma Bank (DSHB) | ADL67.10; RRID: AB_528336 |
| Anti-cGAS (Rabbit monoclonal) | Cell Signaling Technology | Cat# 15102; RRID: AB_2732795 |
| Anti-Phospho-IRF3 (Ser396) (Rabbit monoclonal) | Cell Signaling Technology | Cat# 29047; RRID:AB_2773013 |
| Anti-STING (Rabbit monoclonal) | Cell Signaling Technology | Cat# 13647; RRIDAB_2732796 |
| Anti-IRF3 (Mouse monoclonal) | Developmental Studies Hybridoma Bank | Cat# PCRP-IRF3-1D11; RRID:AB_2618757 |
| Anti-pJNK Thr183/Tyr1850 (Rabbit monoclonal) | Cell Signaling Technology | Cat# 4668; RRID: AB_823588 |
| Anti-pFAK Tyr397 (Rabbit monoclonal) | Thermo Fisher Scientific | Cat# 700255; RRID: AB_2532307 |
| Anti-alpha-tubulin (Mouse monoclonal) | Fisher Scientific | Cat# I50-130-44 |
| Goat anti-Mouse IgG, Alexa594 | Thermo Fisher Scientific | Cat# A-11005;  RRID: AB2534073 |
| Goat anti-Rabbit IgG, Alexa594 | Thermo Fisher Scientific | Cat# A-11012, RRID: AB_ 2534079 |
| IRDye 800CW Goat anti-Rabbit IgG | LI-COR Biosciences | Cat# 926-32211; RRID:AB_621843 |
| IRDye 800CW Goat anti-Mouse IgG | LI-COR Biosciences | Cat# 926-32210; RRID:AB_621842 |
|  |  |  |
| Chemicals, Peptides, and Recombinant Proteins | | |
| 16% paraformaldehyde | Electron Microscopy Sciences | Cat# RT15710 |
| TRIzol™ Reagent | Invitrogen | Cat# 15596026 |
| DAPI | Sigma-Aldrich | Cat# D9542 |
| Vectashield | Vector Laboratories | Cat# H-1000 |
| FITC-conjugated cholera toxin B | Sigma-Aldrich | Cat# C1655 |
| poly(dA:dT) | InvivoGen | Cat# tlrl-patn |
| 2’3’-cGAMP | ApexBio | Cat# BB362 |
| H-151 | Cayman Chemical Company | Cat# 25857 |
| PF573228 | Cayman Chemical Company | Cat# 14924 |
| PF431396 | Cayman Chemical Company | Cat# 17665 |
| Defectinib | Cayman Chemical Company | Cat# 17737 |
| PND1186 | Cayman Chemical Company | Cat# 17668 |
| FAK Inhibitor 14 | Cayman Chemical Company | Cat# 14485 |
| SP600125 | MedChem Express | Cat# HY-12041 |
| CC-401 | MedChem Express | Cat# HY-13022 |
| CC-930 | Cayman Chemical Company | Cat# 22466 |
| Bentamapimod | MedChem Express | Cat# HY-14761 |
| JNK-IN-8 | Cayman Chemical Company | Cat# 15946 |
| GSK 8612 | Cayman Chemical Company | Cat# 28813 |
| BAY-985 | Cayman Chemical Company | Cat# 34234 |
| BMS-345541 | Cayman Chemical Company | Cat# 16667 |
| BAY 11-7082 | Cayman Chemical Company | Cat# 10010266 |
|  |  |  |
| Critical Commercial Assays | | |
| iTaq™ Universal SYBR Green Supermix | Bio-Rad | Cat#1725120 |
| iScript™ Reverse Transcription Supermix for RT-PCR | Bio-Rad | Cat#1708840 |
|  |  |  |
| *D. melanogaster* Strains | | |
| *yw;; FRT82B* | Gift from Dr. Tian Xu (Yale Univ.) | N/A |
| *w*; *UAS-Ras^V12^; FRT82B* | Gift from Dr. Tian Xu (Yale Univ.) | N/A |
| *w*; *UAS-Ras^V12^; FRT82B, scrib^1^ /TM6B* | Gift from Dr. Tian Xu (Yale Univ.) | N/A |
| *yw, ey-Flp1; act>y+>GAL4, UAS-GFP.S65T; FRT82B, tub-GAL80*. | Gift from Dr. Tian Xu (Yale Univ.) | N/A |
| *w^1118^* | Bloomington Drosophila Stock Center | RRID:BDSC_3605 |
| Sco/Cyo, tub-RFP; UAS-Ras^V12^, FRT82B, scrib^1^/Tm6B | Gift from Dr. Andreas Bergmann (UMass Chan Medical School) | N/A |
| *yw, Drs-GFP, Dipt-lacZ* | Bloomington Drosophila Stock Center | RRID:BDSC_55707 |
| *w; Hml****^Δ^****-Gal4, UAS-EGFP* | Bloomington Drosophila Stock Center | RRID:BDSC_30142 |
| *UAS-rpr* | Bloomington Drosophila Stock Center | RRID:BDSC_5824 |
| RNAi of STING: *yv; P{y[+t7.7] v[+t1.8]=TRiP.JF01138}attP2* | Bloomington Drosophila Stock Center | RRID:BDSC_31565 |
| RNAi of STING: *UAS-CG1667RNAi* | National Institute of Genetics (NIG-Fly) | NIG: 1667R-1 |
| RNAi of STING: *UAS-CG1667RNAi* | National Institute of Genetics (NIG-Fly) | NIG: 1667R-3 |
| RNAi of STING: *w; P{GD1905}v4031* | Vienna Drosophila Resource Center (VDRC) | VDRC ID: 4031 |
| RNAi of Bsk: y, sc, sev; P{y[+t7.7] v[+t1.8]=TRiP.HMS04479attP40 | Bloomington Drosophila Stock Center | RRID:BDSC 57035 |
| DN of Bsk: w[1118], P{w[+mC]=UAS-bsk.DN}2 | Bloomington Drosophila Stock Center | RRID: BDSC 6409 |
| RNAi of Fak: y, v; P{y[+t7.7] v[t1.8]=TRiP.HMS02792attP40 | Bloomington Drosophila Stock Center | RRID: BDSC 44075 |
| RNAi of Fak: w; P{KK101680}v108608 | Vienna Drosophila Resource Center (VDRC) | VDRC ID: 108608 |
| RNAi of Rel: *UAS-CG11992RNAi* | National Institute of Genetics (NIG-Fly) | NIG: 11992R-1 |
| RNAi of IKKβ: y, v; P{y[+t7.7] v[+t.18]=TRiP.HMJ23691}attP40/CyO | Bloomington Drosophila Stock Center | RRID: BDSC 62334 |
| RNAi of IKKɛ: P{NIG.2615R}1 | National Institute of Genetics (NIG-Fly) | NIG: 2615R-1 |
| RNAi of IKKɛ: P{KK102408}VIE-260-B | Vienna Drosophila Resource Center | VDRC ID: 103748 |
| RNAi of Dronc: P{NIG.8091R}1 | National Institute of Genetics (NIG-Fly) | NIG: 8091R-1 |
| DN of Dronc: w; P{w[+mC]=UASp-dronc-CARD}5A | Bloomington Drosophila Stock Center | RRID: BDSC 58992 |
| RNAi of Dcp1: w; P{GD10729}v34330 | Vienna Drosophila Resource Center | VDRC ID: 34330 |
| RNAi of Drice: w; P{GD12284}v28065 | Vienna Drosophila Resource Center | VDRC ID: 28065 |
| RNAi of Bub3: y, sc, v, sev; P{y[+t7.7] v[t+1.8]=TRiP.HMS00789]attP2 | Bloomington Drosophila Stock Center | RRID: BDSC 32989 |
| RNAi of Pnut: y, sc, v, sev; P[y[+t7.7] v[t1.8]=TRiP.HMC05924]attP40 | Bloomington Drosophila Stock Center | RRID: BDSC 65157 |
| RNAi of Puc: y, sc, sev; P{y[+t7.7] v[+t1.8]=TRiP.HMS04497}attP40 | Bloomington Drosophila Stock Center | RRID: BDSC 57300 |
| w; P{w[+mC]=UAS-p35.H}BH1 | Bloomington Drosophila Stock Center | RRID: BDSC 5072 |
|  |  |  |
| Human Cell Lines |  |  |
| U87 | ATCC | RRID:CVCL_0022 |
| DU145 | ATCC | RRID:CVCL_0105 |
| MDA-MB-231 | ATCC | RRID:CVCL_0062 |
|  |  |  |
| Oligonucleotides | | |
| Primers for *Diptericin B*:  Forward: 5’- CTCGAGTGCCTGGGCTTATC -3’  Reverse: 5’- AAGGTGCTGGGCATACGATC -3’ | FlyPrimerBank | PD45156 |
| Primers for *Drosomycin*:  Forward: 5’- CTGGGACAACGAGACCTGTC -3’  Reverse: 5’- ATCCTTCGCACCAGCACTTC -3’ | FlyPrimerBank | PD40133 |
| Primers for *Defensin*:  Forward: 5’- CGTGGCTATCGCTTTTGCTC -3’  Reverse: 5’- TTTGAACCCCTTGGCAATGC -3’ | FlyPrimerBank | PD44299 |
| Primers for *Attacin-A*:  Forward: 5’- CCTTGACGCACAGCAACTTC 3’  Reverse: 5’- CCGATCCCGTGAGATCCAAG -3’ | FlyPrimerBank | PD44363 |
| Primers for *Metchinikowin*:  Forward: 5’- ATGCAACTTAATCTTGGAGCGA -3’  Reverse: 5’- GACGGCCTCGTATCGAAAATG -3’ | FlyPrimerBank | PP4078 |
| Primers for *Drosocin*:  Forward: 5’- TTTTCCTGCTGCTTGCTTGC -3’  Reverse: 5’- GGCAGCTTGAGTCAGGTGAT -3’ | FlyPrimerBank | PD40139 |
| Primers for *dSTING (CG1667)*:  Forward: 5’- CACCGTGTTCTTGGCTGATCT -3’  Reverse: 5’- GGGCAAATAGTACCGCCCAT -3’ | FlyPrimerBank | PP21772 |
| Primers for *CG32368:*  Forward: 5’- ATGGAGCTCGAGCACGAAAT -3’  Reverse: 5’- TGCTCCTCGATCATCTTGGC -3’ | FlyPrimerBank | PD40834 |
| Primers for CG*11671*:  Forward: 5’- CTGGCCGACGATAAAAATATCCA-3’  Reverse: 5’- CCTCCGTAAGTCCGTAGGC -3’ | FlyPrimerBank | PP1452 |
| Primers for *Srg1 (CG13641)*:  Forward: 5’-GTGTCCATTATCCGCACAAG-3’  Reverse: 5’-ACTGGGGTATCTGACGGATG-3’ | Holleufer et al., 2021 | ^1^ |
| Primers for *Srg2 (CG42825)*:  Forward: 5’-GCGTTTTGGCCCTTATTATG-3’  Reverse: 5’- CTTTTGTAGCCGACGCAGTG-3’ | Holleufer et al., 2021 | ^1^ |
| Primers for *Srg3 (CG33926)*:  Forward: 5’- GCGACCGTCATTGGATTGG-3’  Reverse: 5’- TGATGGTCCCGTTGATAGCC-3’ | Holleufer et al., 2021 | ^1^ |
| Primers for *RpL32*:  Forward: 5’- GCTAAGCTGTCGCACAAATG-3’  Reverse: 5’- GTTCGATCCGTAACCGATGT-3’ | Kwon et al., 2015 | ^2^ |
| Synthesis of 45-bp interferon stimulatory DNA: Forward: 5’-Cy5-TACAGATCTACTAGTGATCTATGACTGATCTGTACATGATCTACA-3’ Reverse: 5’-TGTAGATCATGTACAGATCAGTCATAGATCACTAGTAGATCTGTA-3’ | Mingjian et al., 2018 | ^3^ |
|  |  |  |
| Software and Algorithms | | |
| Fiji | ImageJ | <http://fiji.sc/> |
| Prism 9.0 | GraphPad | www.graphpad.com |
| Leica Application Suite X (LAS X) software | Leica | RRID:SCR_013673 |

1. Holleufer, A., Winther, K.G., Gad, H.H., Ai, X., Chen, Y., Li, L., Wei, Z., Deng, H., Liu, J., Frederiksen, N.A., et al. (2021). Two cGAS-like receptors induce antiviral immunity in Drosophila. Nature *597*, 114-118. 10.1038/s41586-021-03800-z.

2. Kwon, Y., Song, W., Droujinine, I.A., Hu, Y., Asara, J.M., and Perrimon, N. (2015). Systemic organ wasting induced by localized expression of the secreted insulin/IGF antagonist ImpL2. Dev Cell *33*, 36-46. 10.1016/j.devcel.2015.02.012.

3. Du, M., and Chen, Z.J. (2018). DNA-induced liquid phase condensation of cGAS activates innate immune signaling. Science *361*, 704-709. 10.1126/science.aat1022.
